# CPMI: comprehensive neighborhood-based perturbed mutual information for identifying critical states of complex biological processes

**DOI:** 10.1186/s12859-024-05836-0

**Published:** 2024-06-15

**Authors:** Jing Ren, Peiluan Li, Jinling Yan

**Affiliations:** 1 School of Mathematics and Statistics, Henan University of Science and Technology, Luoyang, 471000 China; 2 Longmen Laboratory, Luoyang, 471003 Henan China; 3 Key Laboratory of Information Fusion Technology of Ministry of Education, School of Automation, Northwestern Polytechnical University, Xi’an, 710072 China

**Keywords:** Tipping point, Dynamic network biomarker (DNB), Transcription factors, Dark genes, Perturbed neighbourhood mutual information (PMI)

## Abstract

**Background:**

There exists a critical transition or tipping point during the complex biological process. Such critical transition is usually accompanied by the catastrophic consequences. Therefore, hunting for the tipping point or critical state is of significant importance to prevent or delay the occurrence of catastrophic consequences. However, predicting critical state based on the high-dimensional small sample data is a difficult problem, especially for single-cell expression data.

**Results:**

In this study, we propose the comprehensive neighbourhood-based perturbed mutual information (CPMI) method to detect the critical states of complex biological processes. The CPMI method takes into account the relationship between genes and neighbours, so as to reduce the noise and enhance the robustness. This method is applied to a simulated dataset and six real datasets, including an influenza dataset, two single-cell expression datasets and three bulk datasets. The method can not only successfully detect the tipping points, but also identify their dynamic network biomarkers (DNBs). In addition, the discovery of transcription factors (TFs) which can regulate DNB genes and nondifferential ‘dark genes’ validates the effectiveness of our method. The numerical simulation verifies that the CPMI method is robust under different noise strengths and is superior to the existing methods on identifying the critical states.

**Conclusions:**

In conclusion, we propose a robust computational method, i.e., CPMI, which is applicable in both the bulk and single cell datasets. The CPMI method holds great potential in providing the early warning signals for complex biological processes and enabling early disease diagnosis.

**Supplementary Information:**

The online version contains supplementary material available at 10.1186/s12859-024-05836-0.

## Background

Complex biological processes are often characterized by abrupt transitions rather than smooth progressions [1–8]. These processes with such a critical transition can generally be categorized into three states [1], which are the normal state, critical state and post-critical state. The normal state represents the initial stage of a biological progression, where the system demonstrates relative normality and high stability. The critical state is a pivotal state in which biological processes undergo abrupt alterations [1, 9]. The post-critical state refers to the stage following the critical state, during which the system enters a new state of balance. A variety of biological processes such as cell fate commitment [10, 11], cell differentiation [12] and disease progression [2] are involved in the transition of critical states. The identification of critical states in biological processes holds significant potential for enhancing our understanding of disease mechanisms and progression. Furthermore, by providing early warning signals, it has the capacity to predict and diagnose diseases before they deteriorate [13]. The detection of critical states can also serve as valuable guidance for disease treatment and intervention. However, it is difficult to detect the critical states directly by conventional biomarkers due to the similarity in clinical phenotype and gene expression profiles between the pre-transition and critical states.

Recently, a novel concept known as dynamic network biomarkers (DNBs) has emerged to capture important transitions in complex biological processes [14]. DNBs distinguish themselves from traditional biomarkers by exhibiting a significant increase in correlation among DNB molecules, while the correlation between DNB and non-DNB molecules diminishes as the system approaches a critical state. Consequently, the DNB approach can be used to detect the critical state of a biological system prior to the transition. To unveil the critical state of complex biological processes at the single-sample level, a sample-perturbed directed network was employed [15]. The Kullback–Leibler divergence (KL) was used to identify the critical state of complex diseases by capturing dynamic changes in multivariable distributions [16]. The Hidden Markov Model (HMM) can detect the early warning signals in the complex biological processes by discerning distinct dynamic characteristics between the pre-critical and post-critical stages [3, 17]. Simultaneously, the extensive utilization of single-cell RNA sequencing (scRNA-seq) data in cell biology and disease research has provided deeper insights into transcriptome features at the individual cell level, facilitating our comprehension of cellular heterogeneity, cell development, and disease mechanisms. Despite the wealth of information contained in single-cell expression data, technical limitations may compromise data quality. Moreover, in contrast to bulk RNA-seq, scRNA-seq is susceptible to higher noise levels and lower coverage, presenting novel computational challenges. Additionally, despite the widespread adoption of the DNB theory, due to the strong noise of the high-dimensional small sample data, the traditional methods still suffer from the effectiveness and robustness problems, especially for single-cell expression data. Thus, it is difficult to develop an effective and robust method to identify the critical states of complex biological processes.

In this study, we propose a novel computational method called CPMI, based on the neighborhood gene correlation network, to detect the tipping point or critical state during a complex biological process. Our method consists of several key steps. Firstly, a network is constructed at each time point through the computation of a modified version of the Mahalanobis distance between gene pairs, thereby assessing the correlation between nodes. Next, the $K$ nearest neighbor genes of the central gene in the local network are selected based on the top $K$ genes in terms of distance. Subsequently, based on reference samples, case samples are separately introduced at each time point, and the perturbed neighbourhood mutual information for the combined samples is calculated, providing insights into changes for each gene at each moment. Finally, we employ the CPMI score to quantify the perturbed information brought by a particular sample or cell relative to a group of given reference samples or cells. The CPMI method provides a reliable approach for identifying the critical states in complex biological processes. The advantages of our proposed CPMI method can be summarized as follows: (i) As a model-free and data-driven method, CPMI is not only suitable for both bulk and single-cell expression data, but also enables individualised diagnosis and treatment based on a single sample of an individual. (ii) The incorporation of gene-neighbour relationships in CPMI effectively reduces the noise and enhances the robustness and effectiveness. (iii) Based on the CPMI method, we can not only detect the critical states during complex biological processes but also identify DNBs. (iv) Through the CPMI method, we discovered nondifferential ‘dark genes’ and uncovered TFs associated with embryonic differentiation.

Applying the CPMI method to a simulated dataset generated by the artificial gene regulatory network, we evaluated its performance compared to the existing mutual information weighted entropy (MIWE) and Jensen-Shannon Divergence (sJSD) method [18, 19] under different noise strengths. The results illustrated that the CPMI method outperformed the aforementioned methods in its ability to detect tipping points and identify DNBs. The CPMI method was applied to six real diseases, including an influenza dataset, two sing-cell datasets and three cancer datasets. By using our method, we succesfully detected the critical states or tipping points within these datasets. In addition, we uncovered transcription factors (TFs) responsible for regulating DNB genes, as well as some ‘dark genes’ that play significant roles in triggering systemic catastrophic consequences. DNBs are defined as the top 5% of genes with the largest CPMI scores at critical state, while ‘dark genes’ are DNBs that do not show differential expression at the molecular level but exhibit high sensitivity to changes in CPMI scores, thus categorizing them as DNBs. Traditional methods often overlook ‘dark genes’, but our approach facilitates their discovery. Furthermore, through functional analysis, we elucidated the potential mechanisms of TFs and ‘dark genes’ in the critical states of complex biological processes.

## Methods

### Theoretical background

A critical state in a complex biological system refers to a transitional stage in system progression which is usually considered to be a pivotal point in disease progression and may have a significant impact on the treatment and prognosis of patients [2]. According to the DNB theory, DNBs are a group of biological molecules that exhibit the following three key changes as a complex system approaches a tipping point [9]: a rapid increase in correlation between members within the DNB group; a sharp increase in variation among DNB group members; and a rapid decrease in correlation between the DNB molecules and any other non-DNB molecules. DNBs can be used to reveal the early warning signals of complex biological processes.

The CPMI method proposed is based on the DNB theory, and we construct the networks in both given reference samples and perturb samples for each time period. In any two local networks, pearson correlation coefficients are calculated for the $\text{K}$ nearest neighbour genes of the central gene, and then the information difference between the two genes is estimated according to the mutual information. The robustness and effectiveness of the method is increased by considering the neighbor genes. By comparing mutual information between the reference and combined samples, the perturbed mutual information is obtained at each time point. Finally, the CPMI quantifies the dynamic changes in individual differences across different time points. The CPMI score is used to detect the critical states of complex biological processes and discover the early warning signals.

## Data progression and functional analysis

The CPMI method proposed in this paper has been applied to six datasets, including an influenza dataset (GEO: GSE30550), human embryonic stem cell to definitive endoderm cells (hESC-to DEC; GEO: GSE75748), mouse ESC to mesoderm progenitor (mESC-to-MP; GEO: GSE79578) from the GEO database (http://www.ncbi.nlm.nih.gov/geo) and kidney renal papillary cell carcinoma (KIRP), colon adenocarcinoma (COAD), thyroid carcinoma (THCA) from The Cancer Genome Atlas (TCGA) database (http://cancergenome.nih.gov).

Identification and prediction of potential upstream transcriptional regulators was based on the online website Chea3 (http://maayanlab.cloud/chea3/). The enrichment analysis of DNBs and transcription factors was conducted through the Gene Ontology Consortium (http://geneontol-ogy.org), Metascape (https://metascape.org/) and DAVID Bioinformatics Resources (https://david.ncifcrf.gov/), while the results of transcription factor enrichment analysis were visualized using Circos (http://www.circos.ca/). The gene function annotation of each dataset was obtained through GeneCards (http://www.genecards.org/). Protein–Protein Interaction (PPI) networks were obtained through the use of the online web page STRING (https://string-db.org/) and visualized using the client software Cytoscape (https://cytoscape.org/).

## Algorithm to identify the critical state based on CPMI

**Fig. 1:**
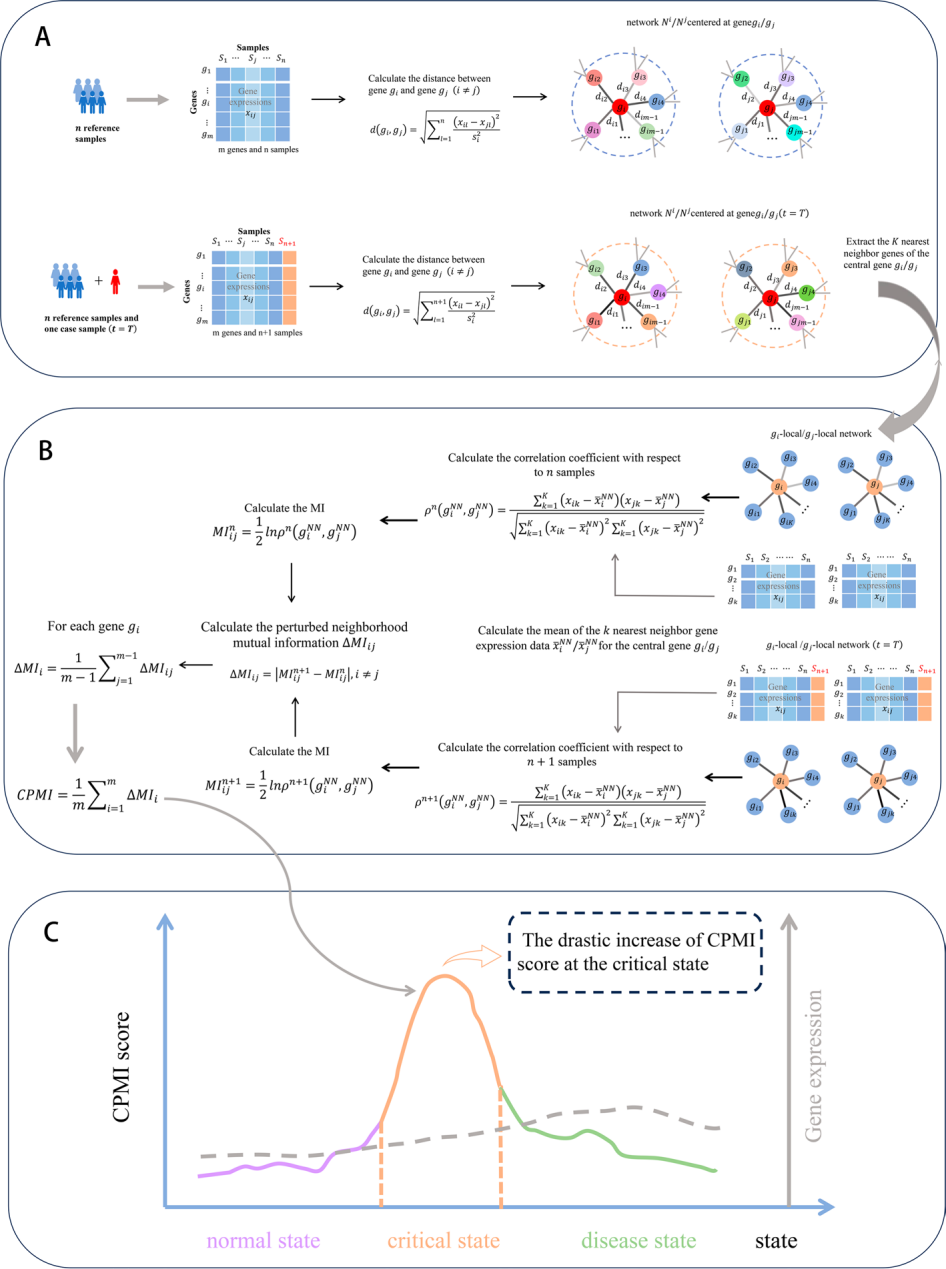
The schematic of the CPMI method for identifying the critical states of complex biological processes. **A** Given a group of control samples and case samples derived at time point $t$, the correlation network is constructed by a modified version of Mahalanobis distance. **B** Extract the $K$ nearest neighbor genes of each gene. Thus, the local network is centered on gene ${g}_{i}\left(i=\text{1,2},\cdots ,m\right)$ and contains other $K$ neighbourhood genes $\left\{{g}_{i1},{g}_{i2},\cdots ,{g}_{iK}\right\}$. Then, the CPMI is calculated for each gene. **C** During the progression of the biological process, The CPMI score increases significantly marking the imminence of a critical state

The CPMI algorithm is a model-free and data-driven method for identifying critical states and DNBs of complex biological processes. Given the reference samples and case samples, we designed the following algorithm to identify the critical state. Figure 1 shows the flowchart schematic of the CPMI algorithm.

[step1] Data processing and sample selection.

Duplicate genes and null values are removed and the gene expression data is normalized. In order to construct the network, a set of molecular sequencing data is utilized, comprising $n$ reference samples and one case sample. For the datasets GSE30550, GSE75748, and GSE79578 obtained from the GEO database, the reference samples are selected from the initial time point of the biological progression, while the case samples represented the samples from other time points. As for the datasets of KIRP, COAD, and THCA obtained from the TCGA database, the reference samples consisted of relatively healthy samples, whereas the case samples consisted of the samples at different stages of cancer development.

[step2] Construst correlation network.

The Mahalanobis distance is used to construct the global network with $n$ reference samples and $n+1$ samples ($n$ reference samples and a case sample), here we used a modified version of the Mahalanobis distance. Calculate the correlation between each two genes in the reference sample, each sample contains $m$ genes, for gene ${g}_{i}\left(i=\text{1,2},\cdots ,m\right)$ and gene ${g}_{j}\left(j=\text{1,2},\cdots ,m\right)$, as defined below

$$d\left( {g_{i} ,g_{j} } \right) = \sqrt {\mathop \sum \limits_{l = 1}^{n} \frac{{\left( {x_{il} - x_{jl} } \right)^{2} }}{{s_{i}^{2} }}} ,\quad { }\left( {i \ne j} \right),$$

where ${x}_{il}/{x}_{jl} \left(l=\text{1,2},\cdots ,n\right)$ is the expression data of the gene ${g}_{i}/{g}_{j}$ in the $l$-th sample, ${s}_{i}$ denotes the standard deviation of the gene ${g}_{i}$ in $n$ samples.

A case sample is added to the $n$ reference samples to form $n+1$ mixed samples, and the correlation between each two genes in the $n+1$ samples is calculated.

$$d\left( {g_{i} ,g_{j} } \right) = \sqrt {\mathop \sum \limits_{l = 1}^{n + 1} \frac{{\left( {x_{il} - x_{jl} } \right)^{2} }}{{s_{i}^{2} }}} ,\quad { }\left( {i \ne j} \right),$$

where ${x}_{il}/{x}_{jl} \left(l=\text{1,2},\cdots ,n,n+1\right)$ is the expression data of the gene ${g}_{i}/{g}_{j}$ in the $l$-th sample, ${s}_{i}$ denotes the standard deviation of the gene ${g}_{i}$ in $n+1$ samples.

[step3] Extract the local network.

After constructing the global network, generating a local network with each gene as the central gene would result in $m$ local networks, where $m$ is the total number of genes. Each local network includes the central gene ${g}_{i}\left(i=\text{1,2},\cdots ,m\right)$ and the other m-1 neighbourhood genes $\left\{{g}_{i1},{g}_{i2},\cdots ,{g}_{im-1}\right\}$. Considering all gene pairs in model construction is an ideal practice, but it may present computational challenges, particularly when dealing with a large number of genes. This not only substantially extends computation time but also escalates computational costs. Therefore, in this paper, we selected $K$ gene pairs to calculate.

The remaining $m-1$ genes excluding the central gene ${g}_{i}$ are arranged in descending order according to distance from the central gene, and the top $K$ genes with the smallest distance are the neighbor genes of the central gene ${g}_{i}$ of the local network${N}_{i}$, denoted as${g}_{i}^{NN}=\left\{{g}_{i1},{\cdots ,g}_{ik},\cdots ,{g}_{iK}\right\}$,$\left(i=\text{1,2},\cdots ,m ,k=\text{1,2},\cdots ,K\right)$.The nearest neighbor genes of the central gene ${g}_{j}$ of the local network${N}_{j}$, denoted as${g}_{j}^{NN}=\left\{{g}_{j1},{\cdots ,g}_{jk},\cdots ,{g}_{jK}\right\}$,$\left(j=\text{1,2},\cdots ,m ,k=\text{1,2},\cdots ,K\right)$.

[step4] Calculate the correlation coefficient to n samples.

For local networks ${N}_{i}$ and ${N}_{j}$, the correlation coefficients of the two sets of nearest neighbour genes based on $n$ reference samples is defined by

$$\rho^{n} \left( {g_{i}^{NN} ,g_{j}^{NN} } \right) = \frac{{\mathop \sum \nolimits_{k = 1}^{K} \left( {x_{ik} - \overline{x}_{i}^{NN} } \right)\left( {x_{jk} - \overline{x}_{j}^{NN} } \right)}}{{\sqrt {\mathop \sum \nolimits_{k = 1}^{K} \left( {x_{ik} - \overline{x}_{i}^{NN} } \right)^{2} \mathop \sum \nolimits_{k = 1}^{K} \left( {x_{jk} - \overline{x}_{j}^{NN} } \right)^{2} } }}{ },$$

where ${x}_{ik}/{x}_{jk}$ is the expression data of the $k$-th nearest neighbour gene ${g}_{ik}/{g}_{jk}$ for the central gene ${g}_{i}/{g}_{j}$ and ${\overline{x} }_{i}^{NN}/{\overline{x} }_{j}^{NN}$ is the mean value of the expression data of the $K$ nearest neighbour genes for the central gene ${g}_{i}/{g}_{j}$.

[step5] Calculate neighborhood mutual information (MI) of with respect to $n$ samples and $n + 1$ samples ($n$ reference samples and a case sample).

The mutual information itself is a measure used to quantify the correlation between two random variables. However, in this paper, we use the change of mutual information to describe the difference between different stages, rather than focusing on mutual information itself. Specifically speaking, we first calculated the mutual information ${MI}_{ij}^{n}$ and ${MI}_{ij}^{n+1}$ at two different stages respectively, and by comparing the mutual information values of these two stages, we can obtain a quantitative result of the information change between them, denoted as $\Delta {MI}_{ij}$. If the difference in mutual information ($\Delta {MI}_{ij}$) is large, it indicates that a significant change has occurred in the network between these two stages.

Then the neighborhood mutual information of gene ${g}_{i}$ and ${g}_{j}$ in $n$ samples is defined as

$${MI}_{ij}^{n}={MI}^{n}\left({g}_{i}^{NN},{g}_{j}^{NN}\right)=\frac{1}{2}ln{\rho }^{n}\left({g}_{i}^{NN},{g}_{j}^{NN}\right).$$

A single sample of cases is added to the $n$ reference samples, repeat the above steps for the mixed $n + 1$ samples and the neighborhood mutual information of gene ${g}_{i}$ and ${g}_{j}$ in $n+1$ samples is

$${MI}_{ij}^{n+1}={MI}^{n+1}\left({g}_{i}^{NN},{g}_{j}^{NN}\right)=\frac{1}{2}ln{\rho }^{n+1}\left({g}_{i}^{NN},{g}_{j}^{NN}\right).$$

[step6] Calculate the comprehensive perturbed mutual information (CPMI).

Calculate the perturbed neighborhood mutual information $\Delta {MI}_{ij}$ of the case sample, defined as follows

$$\Delta {MI}_{ij}=\left|{MI}_{ij}^{n+1}-{MI}_{ij}^{n}\right|.$$

At time point $T$, calculate the comprehensive neighborhood mutual information for each gene ${g}_{i}$

$${\Delta MI}_{i}=\frac{1}{m-1}{\sum }_{j=1}^{m-1}\Delta {MI}_{ij},$$

In the global network, the average perturbed mutual information of $m$ genes for the case sample is

$${\text{CPMI}}_{T}=\frac{1}{m}{\sum }_{i=1}^{m}{\Delta \text{MI}}_{i}.$$

The CPMI score reflects the perturbed mutual information caused by the case sample at each time point. If there is a noticeable increase in the CPMI score, then the moment T is the tipping point for individual disease progression. Furthermore, the top 5% of genes with the highest CPMI scores are recognized as DNBs. Here, we propose neighborhood mutual information to quantify the change in network information at different stages through the change in mutual information. The CPMI primarily characterizes network fluctuations in mutual information of molecules rather than random fluctuations, which is the key to detecting the tipping point. Therefore, the CPMI algorithm plays a crucial role in providing a robust and reliable early warning signal for the critical state.

## Results

Based on the given reference samples and case samples, calculating the CPMI score can be computed with the aforementioned algorithm to identify the critical states in the development of complex biological processes. To demonstrate the computational process and validate the algorithm's effectiveness, we applied the CPMI method to a numerical simulation dataset and six real datasets. These real datasets include an influenza dataset (GEO: GSE30550), single-cell datasets such as hESC-to-DEC data (GEO: GSE75748) and mESC-to-MP data (GEO: GSE79578) from the NCBI GEO database, as well as bulk data including KIRP, COAD and THCA from The Cancer Genome Atlas (TCGA) database. The successful identification of critical states for all datasets confirms the effectiveness of the CPMI method in identifying the critical state.

## Validation based on numerical simulation

To verify the accuracy of the CPMI algorithm in detecting the tipping point or critical state, we used a 9-node modulated network to generate simulated data to illustrate how the algorithm detects early warning signals. This regulatory network with nine nodes is composed of a set of stochastic differential equations in the Michaelis–Menten equation. Equations in the form of Mikelis–Menten are used to describe the interactions between nodes in a gene regulatory network [20, 21]. These equations are commonly used to study gene regulatory activities [22, 23] such as transcription, translation and nonlinear biological processes [24, 25]. They can also be used to model dynamic behavior in gene regulatory networks, including the gene expression regulation and signaling. Based on the parameter $p$ varies from − 0.5 to 0.23, with $p=0$ representing the bifurcation point, thereby generating the numerical simulation dataset.

**Fig. 2:**
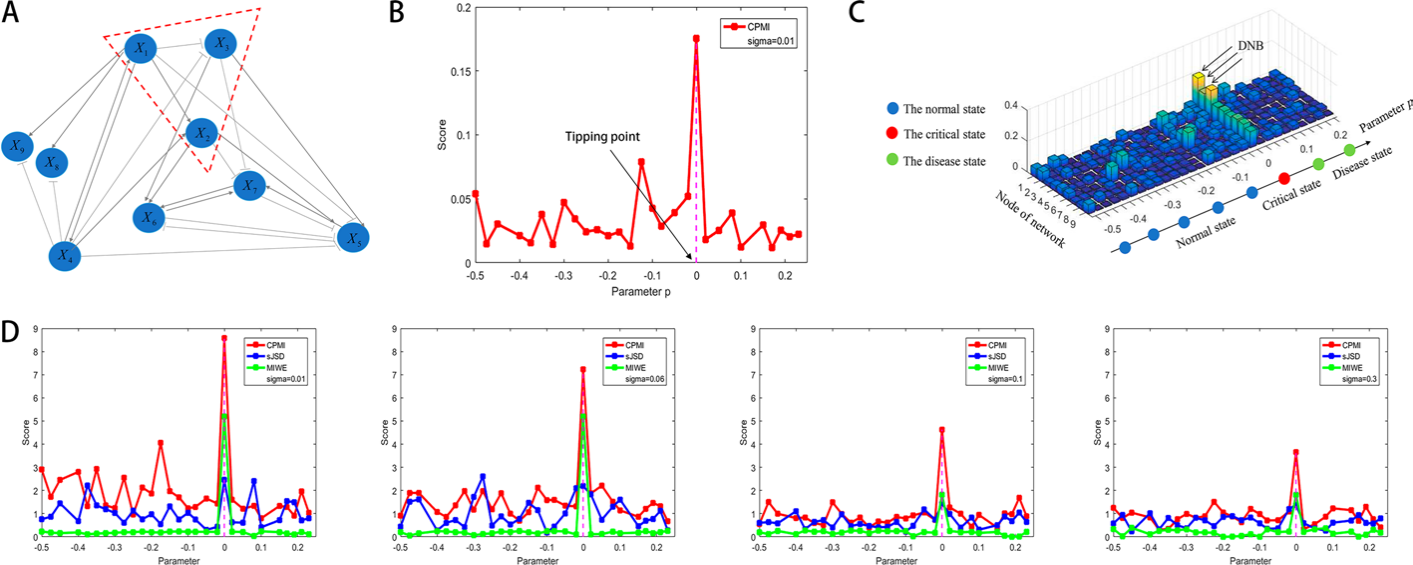
The validation of the CPMI method on a simulation dataset. **A** The model of an 9-node regulatory network, in which the arrow represents positive regulation whereas the blunt line denotes negative regulation.** B** The curve of the CPMI score of the global network. **C** The landscape of the CPMI scores for 9 nodes. **D** Comparison of the robustness between the CPMI method and MIWE, sJSD method at different noise strength

The gene regulatory network with nine nodes is shown in Fig. 2A. The data generated using the initial value of parameter $p$ is used as the reference sample for the numerical simulation. In Fig. 2B, there is a significant increase in the CPMI score, indicating the imminent arrival of a tipping point or critical state when the system approaches $p=0$, which serves as the bifurcation point. Figure 2C illustrates the different dynamics of the system between the normal and critical states by showing the evolution of the CPMI landscape for the 9 nodes. When the parameter $p$ is far from the bifurcation point $p=0$, the CPMI scores of each node remain low and stable. However, as the parameter $p$ tends to 0, the CPMI scores of certain nodes, namely the DNB genes, exhibit a substantial increase, signaling the impending arrival of the critical state.

In addition, simulation experiments were conducted at different noise strengths to compare the performance of the CPMI method with the MIWE [18] and sJSD [19] methods. As the noise strength increases, the CPMI method consistently detects the critical state accurately. Figure 2D demonstrates that the CPMI method outperforms the MIWE and sJSD methods in identifying the critical state under different noise strengths, which proves the effectiveness and robustness of the CPMI method. Further details of the numerical simulation calculations can be found in the Supplementary Material A.

## Identifying the critical state of individual influenza infection

In this study, we applied the CPMI method to the time-series dataset GSE30550 [26] concerning influenza infection. The dataset encompasses samples from 17 volunteers who were infected with the H3N2/Wisconsin virus through intranasal administration. Out of these, nine subjects (including subjects 1, 5, 6, 7, 8, 10, 12, 13, and 15) subsequently developed severe symptoms of infection, while the other eight subjects remained asymptomatic. In subsequent analytical studies, the samples from volunteers with severe influenza-like symptoms were designated as symptomatic infections, and those from the healthy volunteers were classified as asymptomatic infections [11]. Following this classification, the 17 subjects were divided into two groups: the asymptomatic (AS) group and the symptomatic (S) group. Additionally, the dataset is generated based on microarray chip technology, not RNA-seq data.

This dataset comprises 17 subjects with 16 time points, where each individual has only one sample data per time point. However, there are missing samples for certain subjects: the samples from 8th subject at the 21th h, the 13th subject at the − 24th and 36th h, and the 17th subject at the 36th h. The other volunteers have 16 sampling time points (− 24, 0, 5, 12, 21, 29, 36, 45, 53, 60, 69, 77, 84, 93, 101, 108 h), totaling 268 pairs of gene expression data. For the reference samples, we used the gene expression profiles of the first two time points (− 24 and 0 h), while the case samples consisted of the gene expression profiles from the remaining time points for each individual.

**Fig. 3:**
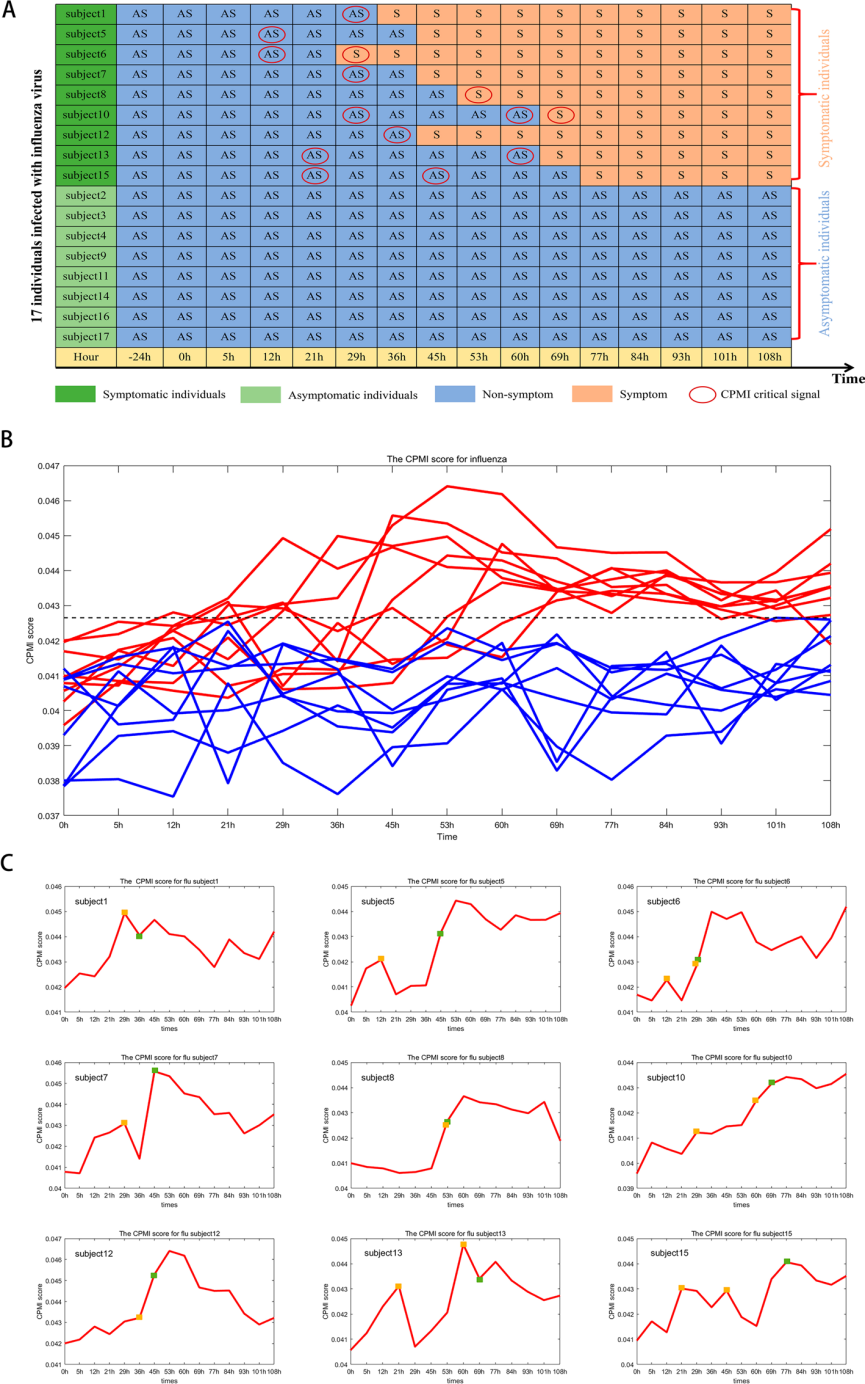
The identifification of the tipping point for influenza infection based on CPMI. **A** The temporal table of the time to the occurrence of influenza symptoms and the tipping point identified by CPMI for all individuals. **B** The CPMI score curve for all 17 subjects. The red curve represents the CPMI scores for nine symptomatic subjects. The blue curve represents the CPMI score for eight asymptomatic subjects.** C** The curves of the CPMI scores for nine symptomatic individuals. The green box represents the initial time to the appearance of influenza symptoms (clinical observation), and the orange box represents the critical state determined by the CPMI score

The results presented in Fig. 3A demonstrate the detection of early warning signals preceding the onset of influenza symptoms in the nine symptomatic subjects. Figure 3B shows the CPMI scores of all subjects at each time point. For subjects with influenza symptoms (red curves), the CPMI scores exhibit a significant increase prior to the influenza symptom onset, thereby providing the early warning signals for imminent critical transitions. In contrast, asymptomatic subjects show no notable change in CPMI scores (blue curves). Furthermore, Fig. 3C presents CPMI scores specifically for the nine symptomatic subjects. The abrupt increase in CPMI scores at the critical state indicates the CPMI algorithm can accurately identify the critical state of an individual. Notably, two warning signals have been detected before the onset of flu symptoms for the 6th, 10th, 13th and 15th subjects. The accurate identification of the critical states for each individual validates the effectiveness of the CPMI method.

## Identifying cell fate commitment during embryonic differentiation

We applied the CPMI method to two cell differentiation time course datasets: hESCs to DECs and mESC-to-MP. The hESCs-DECs dataset is a dataset for studying the differentiation of human embryonic stem cells (hESCs) into definitive endoderm cells (DECs) [27]. This dataset includes gene expression data, phenotype data, and other relevant information collected at different time points. The dataset consists of 758 cells, with 6 time points: 0 h with 92 cell samples, 12 h with 148 cell samples, 24 h with 112 cell samples, 36 h with 218 cell samples, 72 h with 184 cell samples, and 96 h with 234 cell samples. The mESC-to-MP refers to the differentiation process of mouse embryonic stem cells (mESCs) into midbrain progenitors (MP) [11]. This dataset consists of 584 cells, with 4 time points: 0 h with 82 cell samples, 12 h with 168 cell samples, 24 h with 171 cell samples, and 48 h with 164 cell samples.

Following the algorithmic process detailed earlier, we calculated the CPMI score at each time point. In this study, the reference samples are derived from the gene expression profiles of cell samples at the initial time point of the cell differentiation process, while the case samples at different time points correspond to the gene expression profiles of cell samples at each respective time point. The effectiveness and accuracy of CPMI has been validated by successfully detecting cell fate transitions during embryonic cell differentiation. The results of the hESC-to-DEC dataset are detailed in the main text, while the results of the mESC-to-MP dataset are provided in the Supplementary Material B.

## Critical state of hESC-to- DEC

**Fig. 4:**
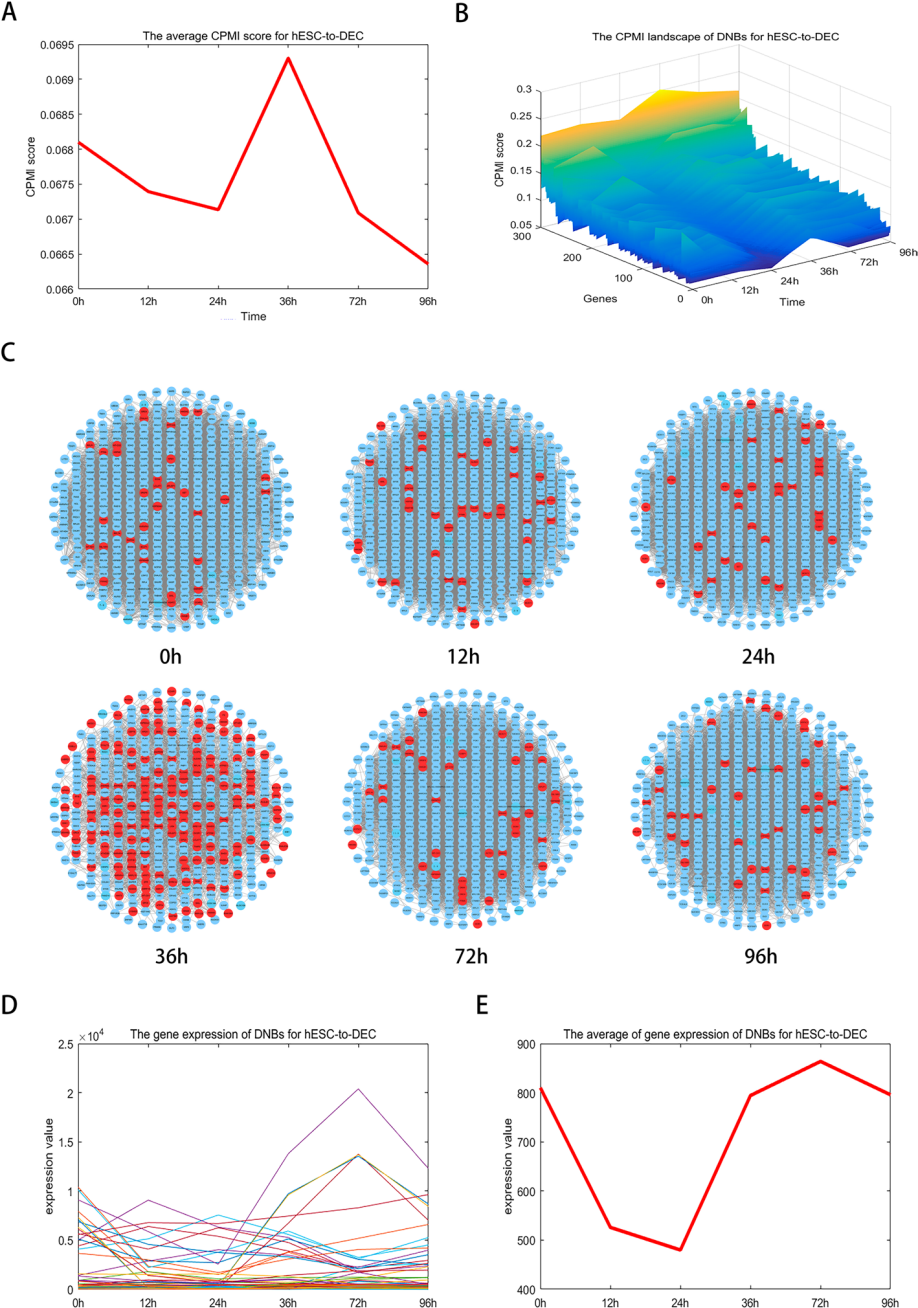
Identification of the cell fate transition of cell differentiation in hESC-to-DEC dataset. **A** The CPMI score curve of hESC-to-DEC. The significant increase of CPMI score at 36 h indicates the arrival of the critical state. **B** The CPMI landscape of DNBs for hESC-to-DEC. The overall CPMI score for the DNB gene is significantly higher at 36 h than at other time points, indicating that it is in a critical state. **C** The dynamic evolution of DNBs for hESC-to-DEC. At 36 h, a significant change in the network shows that a critical warning signal can be detected. **D** The gene expression of DNBs for hESC-to-DEC. **E** The average of gene expression of DNBs for hESC-to- DEC

Diseases related to cell differentiation may result from changes in gene expression, signaling pathways, or epigenetics. Understanding these underlying mechanisms is crucial for better grasping the development and progression of such diseases and for generating new ideas for therapeutic approaches. For the hESC-to-DEC dataset, the red curve in Fig. 4A shows a sharp increase in the CPMI score at 36 h, indicating an upcoming shift in cell fate. An early warning signal for the impending cell fate transition is observed at 36 h. Indeed, the induction of differentiation at 72 h for definitive endoderm (DE) has been previously documented [27]. The top 5% of genes with the highest CPMI scores in the critical state are identified as DNBs, and the landscape evolution of these DNBs is shown in Fig. 4B, demonstrating a significant increase in critical state scores during cell differentiation. The dynamic changes in the DNB genes for the hESC-to-DEC dataset are illustrated in Fig. 4C, with significant alterations in network structure at 36 h. Furthermore, as shown in Fig. 4D and E, the gene expression-based method for DNB genes fails to accurately distinguish the critical phase from other phases. However, utilizing the CPMI scores of these DNB genes enables the accurate identification of the critical state at 36 h.

## Revealing nondifferential ‘dark genes’ and functional analysis for hESC-to-DEC

Within the set of DNBs, there are genes that do not show differential expression at the molecular level but are highly sensitive to changes in the CPMI score. We refer to these genes as ‘dark genes’, and their analysis provides insights into the the important roles that some genes play during embryonic development. The following are the ‘dark gene’ analyses for hESC-to-DEC.

**Fig. 5:**
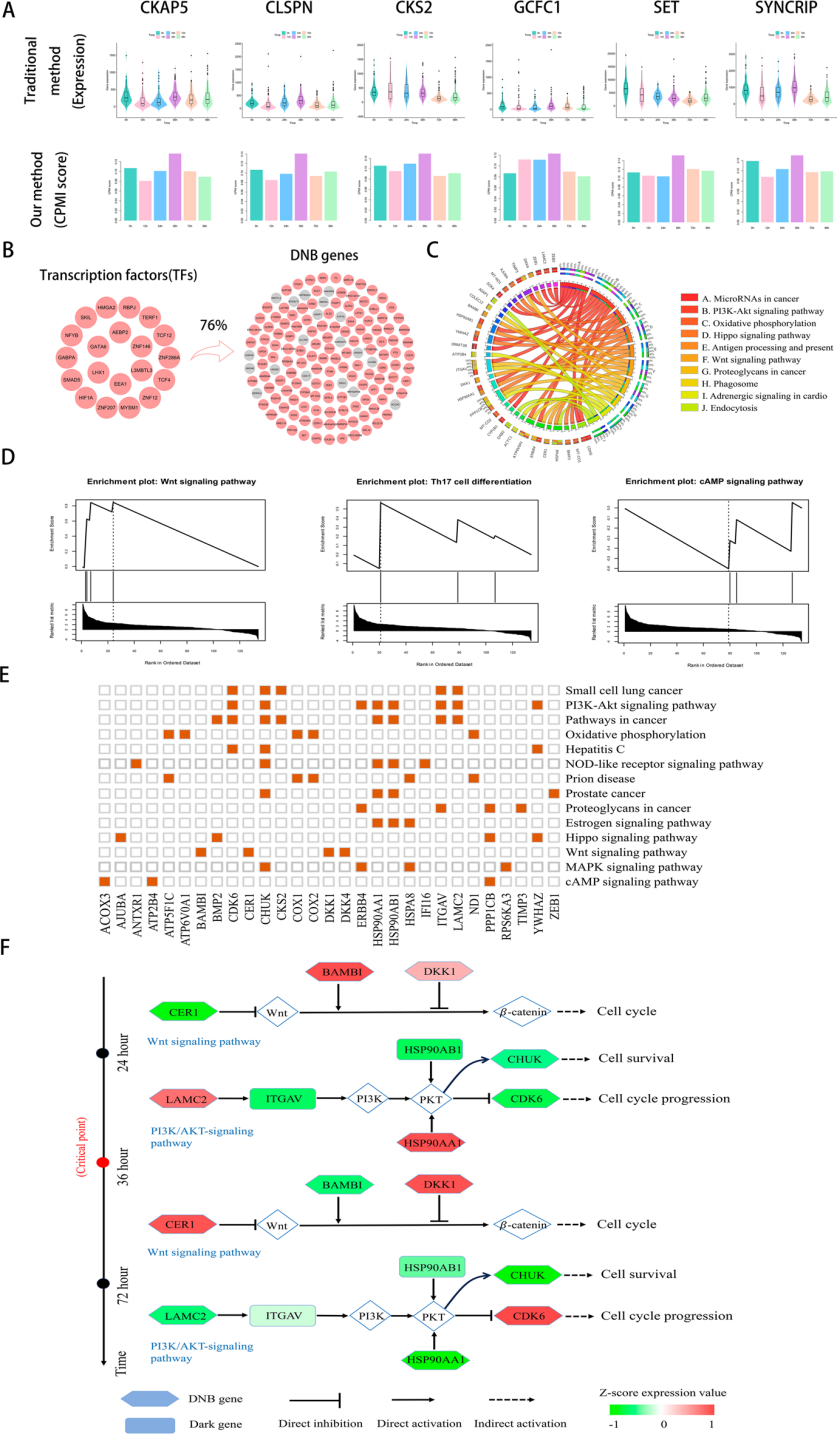
Regulatory mechanisms of embryo development revealed by the DNB genes. **A** The comparison of gene expression and CPMI score of ‘dark genes’ for hESC-to DEC, CKAP5, CLSPN, HSP90AB1, ITGAV, SET and SYNCRIP were found to be ‘dark genes’ of hESC-to-DEC, whose CPMI scores were more sensitive to the early warning signal of embryonic differentiation. **B** The top 20 hub upstream TFs, which regulates 76% of the DNBs that were identified at 36h. **C** KEGG pathway enrichment analysis of regulated DNBs during the hESC-to-DEC process. The left side of the outer ring represents DNBs detected by CPMI algorithm and the right side of the outer ring represent detailed pathway in which these genes are involved. In the inner ring, the color and width of links respectively indicate diverse enrichment pathway and signifificant levels of genes function. **D** GSEA enrichment analysis of ‘dark genes’. The enriched pathways included Wnt signaling pathway, Th17 cell differentiation and cAMP signaling pathway. **E** DNBs are involved in important biological processes and KEGG pathways in hESC-to-DEC process. **F** For the hESC-to-DEC dataset, switching dynamics before and after critical point induced by other DNBs and ‘dark genes’

For hESC-to-DEC dataset, CKAP5, CLSPN, HSP90AB1, ITGAV, SET and SYNCRIP were identified as ‘dark genes’, Fig. 5A visually compares the gene expression data with the corresponding CPMI scores for these ‘dark genes’. Notably, the CPMI score of ‘dark genes’ become more sensitive as the cell fate transition approaches, exhibiting significant upward trends before differentiation into definite endoderm (DE) at 72 h. In comparison to the gene expression data, the CPMI scores for CKAP5 and CLSPN genes exhibit a significant increase at 36 h, corresponding to a critical state in hESC-to-DEC. However, there is no apparent change in gene expression at all times. These findings highlight the effectiveness of the CPMI algorithm in providing early warning signals for impending cell fate transitions. In addition, ‘dark genes’ play significant functional roles in cell differentiation. For example, increased expression of CKAP5 promotes cell proliferation and migration, making it an important prognostic marker [28]. HSP90AB1 is involved in multiple signalling pathways and shows high expression levels in various diseases [29]. In addition, DNMT3B is essential for regulating placental development and function during embryogenesis, which is critical for embryo survival [30].

In order to investigate the mechanism of action of DNBs, we conducted an analysis to identify potential upstream transcriptional regulators (TFs) of DNB genes [31]. TFs are involved in regulating the expression of multiple genes, and they play a key role in the regulation of cell development, differentiation, and defifine the cell identity and drive cell-fate transitions [32, 33]. In this study, the top 20 TFs were identified associated with the DNB genes. As shown in Fig. 5B, our analysis of the hESC-to-DEC dataset reveal that these TFs have the ability to regulate 76% of the DNB genes at the tipping point. In the context of hESCs, ZNF207 collaborates with master pluripotency TFs to govern self-renewal and pluripotency, while also exerting control over cell commitment towards ectoderm through direct regulation of neuronal TFs. Consequently, a distinct isoform of ZNF207 operates at the nexus that balances pluripotency and differentiation to ectoderm in hESCs [34].

In addition, we conducted enrichment analysis of the regulated DNBs, as shown in Fig. 5C, These DNBs demonstrate enrichment in pathways associated with cell differentiation, including MicroRNAs in cancer (hsa05206), PI3K-Akt signaling pathway (hsa04151) and Hippo signaling pathway (hsa04390). The enrichment analysis highlights the involvement of these DNBs in the following biological processes such as negative regulation of transcription from RNA polymerase II promoter (GO:0000122), negative regulation of apoptotic process (GO:0043066), and positive regulation of tau-protein kinase activity (GO:1902949), which further underscores their significance during the cell differentiation from hESCs to DECs.

To reveal the regulatory mechanisms of embryo development revealed by ‘dark genes’ in hESC-to-DEC process, we conducted gene set enrichment analysis (GSEA) using the online platform. The detailed pathway information from the enrichment analysis can be found in Supplementary Figure S5. As shown in Fig. 5D, the enriched pathways primarily include the Wnt signaling pathway, Th17 cell differentiation and cAMP signaling pathway. The Wnt signalling pathway is involved in tissue development and homeostasis, playing a vital role in various functions during embryonic developmental stages, such as stem cell pool regulation, cell migration and specialisation [35]. Th17 cells are central to the pathogenesis of autoimmune and inflammatory diseases, and are important for exploring potential therapeutic targets [36]. Figure 5E demonstrates the important pathways involved in the DNBs, which are closely related to embryo development. For instance, the Estrogen regulates cell proliferation and differentiation through interactions with two different receptors [37]. The Hippo signalling pathway regulates cell proliferation and controls cell cycle, apoptosis and cell differentiation processes [38, 39]. Figure 5F depicts the underlying mechanisms unveiled by the functional analysis of both the other DNBs and ‘dark genes’ in the hESC-to-DEC dataset. In the PIK3/Akt signaling pathway, the downregulation of LAMC2 and upregulation of ITGAV can induce PI3K enzyme activation and promote phosphatidylinositol triphosphate (PIP3) production via Integrin $\upalpha$ V $\upbeta$ 3 binding to Laminin, which in turn inhibits cell migration and proliferation [40]. Moreover, the downregulation of CHUK expression levels may impact the regulatory role of NF-κB in processes such as cell survival and inflammation [41]. CDK6 regulates the cell cycle process, and upregulation of its expression level enhances the rate of cell proliferation,, leading to cell division and growth [42].

## Identifying the critical state during cancer progression

Based on the CPMI method, we identified critical states of KIRP, COAD, and THCA in three cancers using data from the TCGA database. Each disease dataset can be obtained from TCGA for both tumour samples and tumour adjacent samples, and the three cancer case samples were divided into different stages based on clinical information. The results for KIRP are presented in the main text, while the findings for THCA and COAD can be found in the Supplementary Material B.

## Critical state of KIRP

**Fig. 6:**
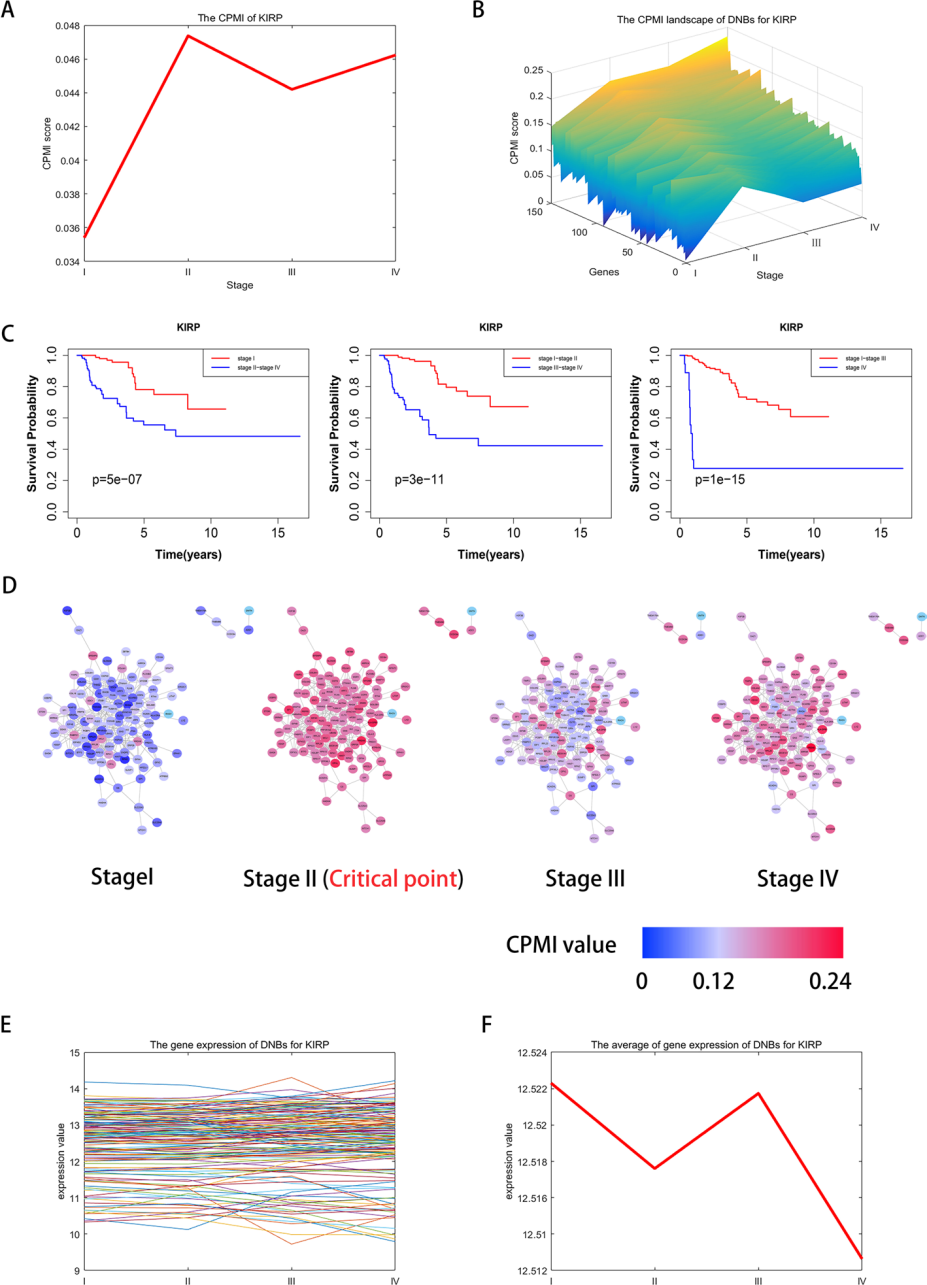
Identification of critical stage for KIRP. **A** The CPMI score curve of KIRP. The score reached its peak at the critical state stage II. **B** The CPMI landscape of DNBs for KIRP. DNBs scores increased significantly at stage II. **C** The comparison of KIRP survival times before and after every state. **D** The change of the DNBs CPMI values for KIRP. The dynamic evolution of the DNBs at different stages is shown, indicating that the critical state is at stageII. **E** The gene expression of DNBs for KIRP. **F** The average of gene expression of DNBs for KIRP

The KIRP dataset from the TCGA database includes 35 tumor-adjacent samples and 270 tumor samples. In Fig. 6A, the KIRP dataset successfully identifies the tipping points or critical states at stage II, indicating irreversible changes in the disease progression. Specifically speaking, the CPMI score shows a rapid increase from stage I to stage II, indicating that the critical state occurs prior to the worsening of the disease, which consistent with the observation that the cancer worsens in stage III. Figure 6B demonstrates the changes in CPMI scores for protein–protein interaction network maps constructed using DNB genes. The CPMI values of these DNB genes vary according to the time period, and unlike other stages, the network diagram of stage II shows significant changes in CPMI values, which suggests the imminent arrival of a tipping point. This showcases the accuracy and effectiveness of our method in detecting the critical ststes before the disease deteriorates, enabling timely and appropriate treatment for patients. As shown in Fig. 6C, we analyzed the prognosis of samples grouped before and after each state. Notably, pre-stage II samples have significantly longer survival times and higher survival probabilities compared to post-stage II samples. In addition, the $\text{p}$-values obtained from the KIRP prognosis analysis were 5e-07 (Stage I), 3e-11 (Stage I-II), and 1e-15 (Stage I-III), all less than 0.05, emphasizing the importance of prognostic analyses.

Figure 6D illustrates the changes in CPMI scores for DNBs at each stage, accurately identifying the critical state as being stage II. Tumour deterioration encompassing local invasion, metastasis, and complications, is a major contributor to cancer-related deaths. Malignant tumor cells possess the ability to invade nearby tissues and organs, as well as spread to distant tissues and organs through the bloodstream or lymphatic system to form distant metastase [43]. Therefore, it is pivotal to determine the critical state of the disease before tumor cell metastasis and diffusion to control tumor deterioration. This enables timely implementation of appropriate treatment strategies to inhibit cancer metastasis and improve treatment outcomes. Through the early warning signals provided by CPMI, clinicians can detect the inflammatory response of the nervous system associated with the tumor in its early stages, allowing for prompt intervention with suitable measures. As shown in Fig. 6E and F, while DNB gene expression alone cannot differentiate between the critical state and other states, our method can accurately identify the critical state.

## Revealing nondifferential ‘dark genes’ and functional analysis for KIRP

**Fig. 7:**
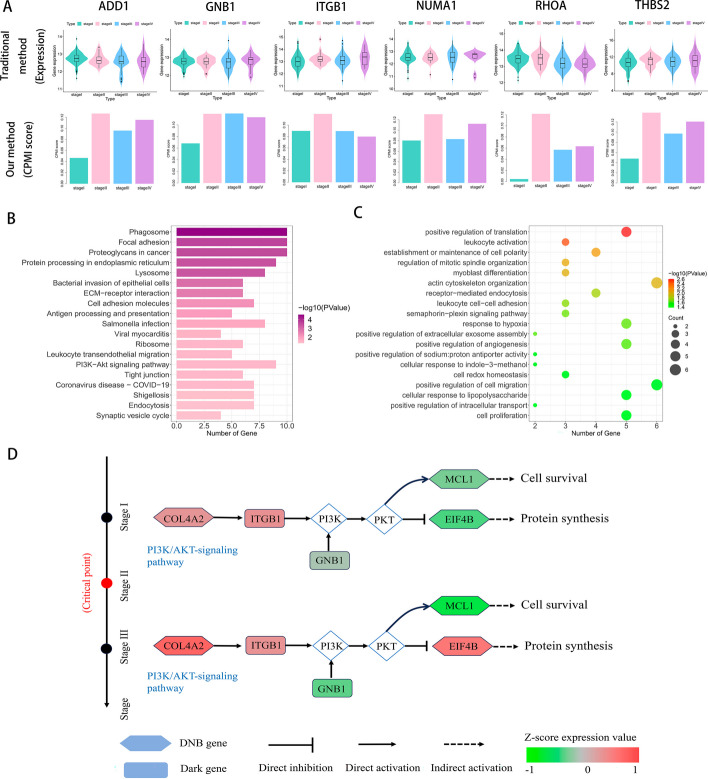
**A** The comparison of gene expression and CPMI score of ‘dark genes’ for KIRP, ADD1, GNB1, ITGB1, NUMA1, RHOA and THBS2, are found to be ‘dark genes’ of KIRP, whose CPMI scores are more sensitive to the early warning signal of disease deterioration. **B** GO analysis shows that DNB genes are involved in several biological processes associated with cancer. **C** Results of KEGG pathway enrichment analysis of DNB genes. **D** For the KIRP dataset, the underlying signaling mechanisms revealed by ‘dark genes’ and DNB genes

‘Dark genes’ are genes that show no significant differences in gene expression levels but are sensitive to CPMI scores. In Fig. 7A, ADD1, GNB1, ITGB, NUMA1, RHOA and THBS2 were identified as ‘dark genes’. When the critical state leading to tumor deterioration arrives, the CPMI scores of these genes become more sensitive and show clear upward trends compared to the gene expression data. For example, the CPMI scores for the ITGB1 gene significantly increased at stage II, the critical state of KIRP, while no noticeable changes in gene expression were observed at any other stage. Similar results were obtained for other ‘dark genes’, which illustrates the accuracy of the CPMI algorithm in capturing the early warning signals of disease deterioration.

In addition, we also discovered that these ‘dark genes’ have significant biological implications in cancer processes. For instance, overexpression of GNB1 in cancer is associated with poor patient prognosis and disease deterioration, as GNB1 activates the PI3K/MAPK signaling pathway to promote hepatocellular carcinoma progression [44]. In gastric cancer, ITGB1 expression correlates with the activity of the Wnt/beta-catenin signalling pathway and can serve as a prognostic marker for various cancers, such as esophageal adenocarcinoma and lung adenocarcinoma [45]. Dysregulated activity of RHOA has been related to the growth, progression and metastasis of various cancer types, positioning RHOA as a crucial regulator and potential therapeutic target [46]. High THBS2 expression is linked to poor patient response to immunotherapy and shorter survival after treatment [47].

Functional analysis was performed on the identified DNBs. The analysis revealed enrichment of DNBs in the PI3K-Akt signaling pathway, Protein processing in endoplasmic reticulum and other cancer-related pathways (Fig. 7B). In particular, proteoglycans in cancer play a significant role in cancer angiogenesis, proliferation, invasion, and metastasis, thereby impacting the progression of the disease [48]. In addition, DNBs were enriched in positive regulation of translation, semaphorin-plexin signaling pathway, positive regulation of extracellular exosome assembly, and other important biological processes crucial for disease development (Fig. 7C). For instance, Semaphorin-Plexin signaling is essential for various cellular aspects of organogenesis, including cell migration, proliferation and survival [49]. Receiver mediated endocytosis is a crucial pathway involved in regulating tumor metastasis and invasion [50]. Moreover, instability in cellular redox homeostasis can lead to gene mutations, thereby promoting cancer development [51].

Figure 7D shows the potential mechanisms revealed through functional analysis of the other DNBs and ‘dark genes’ in the KIRP dataset. Among these genes, the COL1A2 gene is a crucial component involved in supporting and connecting tissues within the extracellular matrix. Upregulation of COL1A2 alters the extracellular matrix environment, thereby facilitating the proliferation, migration, and invasion of cancer cells [52]. ITGB1, an upstream regulator, remains relatively unchanged at the gene expression level. GNB1 participates in the regulation of cellular signal transduction. Upregulated expression of GNB1, in coordination with ITGB1, activates the phosphatidylinositol 3-kinase (PI3K) and AKT/protein kinase pathways, subsequently leading to the downregulation of the MCL1 gene expression. MCL1 is a protein with anti-apoptotic function and is involved in regulating the process of cell survival and apoptosis. Abnormal function or overexpression of MCL1 enhances the anti-apoptotic capacity of cells, thus promoting tumour growth and drug resistance [53]. In addition, after the critical state, the expression level of the EIF4B gene is significantly upregulated, indicating increased synthesis of specific proteins involved in cellular physiological and pathological processes. This contributes to the proliferation and transformation of tumor cells. The collective action of these genes leads to dysregulation of the PI3K/AKT signaling pathway [54], contributing to the development of various diseases such as cancer, diabetes and cardiovascular disease.

## Discussion

Identifying the critical states of complex biological processes is an important and biologically significant task. However, traditional methods applied to high-dimensional small-sample data with strong noise still suffer from the effectiveness and robustness problems, especially for single-cell expression data. Therefore, identifying the critical states of complex biological processes remains a challenging problem. To address this problem, we have developed a novel method, i.e., the CPMI method, to detect the early warning signals of complex biological processes. The CPMI method introduces neighbor mutual information to estimate the difference in information between two genes and quantifies the perturbation mutual information caused by the given reference samples in relation to case samples at each moment, so as to improves the reliability and validity of the early warning signals.

By applying the CPMI method to an influenza dataset, two single-cell expression datasets and three bulk datasets, we can accurately identify the pre-deterioration stage of tumor disease and cell fate commitment during embryonic development. For influenza, the data are derived from the same individual, we can use the CPMI method to achieve individualised disease prediction based on a single sample of an individual. Additionally, the identification of DNBs reveals the potential molecular mechanisms involved in complex biological processes, including disease progression and cell differentiation. Functional analysis further elucidates the important pathways and biological processes associated with these DNB genes. Notably, the discovery of ‘dark genes’, which play a crucial role in complex biological processes and disease prognosis, has provided significant insights. Moreover, the effectiveness and robustness of the CPMI method are verified through numerical simulations conducted under different noise strengths.

## Conclusions

In conclusion, we propose a robust computational method, i.e., CPMI, which is applicable in both the bulk and single cell datasets. The CPMI method holds great potential in providing the early warning signals for complex biological processes and enabling early disease diagnosis.

### Supplementary Information

Supplementary Material: A, B

## Data Availability

The identifiers for the influenza dataset are GEO: GSE30550, for the hESC-to-DEC dataset are GEO: GSE75748, and for the mESC-to-MP dataset are GEO: GSE79578. These three datasets, which support the conclusions of this article, can be downloaded from the NCBI GEO database (http://www.ncbi.nlm.nih.gov/geo/). The identifiers for the kidney renal papillary cell carcinoma dataset are TCGA-KIRP, for the colon adenocarcinoma dataset are TCGA- COAD, and for the thyroid carcinoma dataset are TCGA-THCA. These three datasets, which support the conclusions of this article, can be downloaded from the TCGA repositories (https://portal.gdc.cancer.gov/). To ensure reproducible results, the original code is available at https://github.com/R-J-06/CPMI.
