# Supplementary material for: CPMI: comprehensive neighborhood-based perturbed mutual information for identifying critical states of complex biological processes
Source: BMC Bioinformatics. 2024 Jun 15;25:215. doi: 10.1186/s12859-024-05836-0 (PMC11180411; doi:10.1186/s12859-024-05836-0)
Supplement: Supplementary file 1 — Supplementary Material: A, B [file 12859_2024_5836_MOESM1_ESM.pdf]

Supplementary Material: Comprehensive neighbourhood-based perturbed mutual information for identifying critical states of complex biological systems

Contents

A. The details of numerical simulation S2

B. Critical state of mESC-MP S5

C. Critical state of THCA and COAD S7

D. The analysis of 'dark genes' for hESC-to-DEC and KIRP S9

E. The analysis of 'dark genes' for mESC-to-MP S10

E1. The expression analysis of "dark genes" for mESC-to-MP S10

E2. The functional analysis of "dark genes" for mESC-to-MP S11

F. The functional analysis for hESC-to-DEC and mESC-to-MP S12

F1. The functional analysis for hESC-to-DEC S12

F2. The functional analysis for mESC-to-MP S13

G. Validating the identified critical state by The Kaplan-Meier (log-rank) survival analysis S14

G1. Validating the identified critical state for THCA S14

G2. Validating the identified critical state for COAD S14

H. The functional analysis for THCA and COAD S15

H1. The functional analysis for THCA S15

H2. The functional analysis for COAD S15

I. Leave-one-out cross validation S16

J. The discussion on the Mahalanobis distance denominator S17

References S19

A. The details of numerical simulation

A regulatory network with 9 genes (Figure S1) was used to conduct a numerical simulation for detecting the pre-disease state using CPMI. Such molecular regulatory networks are often used to study various gene regulatory activities including transcription and translation [1,2], and cyclic reaction [3] and nonlinear biological processes [4,5,6]. The following 9 differential equations represent the gene regulations of 9 genes in the network. In the network, gene regulation is represented in a Michaelis-Menten form with the degradation rates, which are linearly proportional to the concentrations of the corresponding genes.

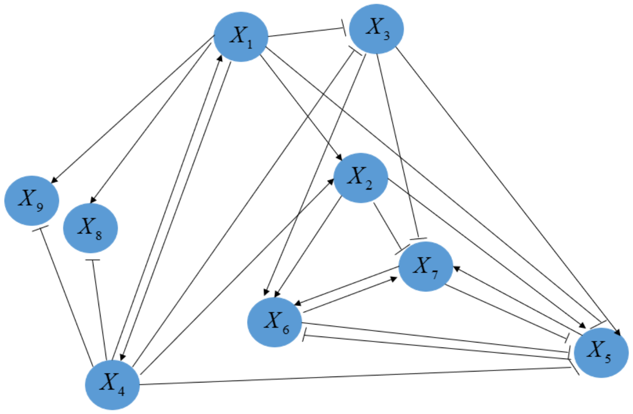

Figure S1. A model of an nine-molecular network. In this sketch of a molecular network, there are nine nodes whose dynamical regulatory relationships are given as stochastic system Eq.(S1). The edges represent positive or negative regulations among nodes.

$$\begin{aligned}
\frac{dz_1(t)}{dt} &= \frac{(10-3|P|)z_4(t)}{20(1+z_4(t))} - \frac{10+3|P|}{20}z_1(t) + \xi_1(t) \\
\frac{dz_2(t)}{dt} &= \frac{(6-3|P|)z_1(t)}{20(1+z_1(t))} + \frac{(6-3|P|)z_4(t)}{20(1+z_4(t))} - \frac{3}{5}z_2(t) + \xi_2(t) \\
\frac{dz_3(t)}{dt} &= \frac{3|P|-8}{10} + \frac{8-3|P|}{20(1+z_1(t))} + \frac{8-3|P|}{20(1+z_4(t))} - \frac{4}{5}z_3(t) + \xi_3(t) \\
\frac{dz_4(t)}{dt} &= \frac{(10-3|P|)z_1(t)}{20(1+z_1(t))} - \frac{10+3|P|}{20}z_4(t) + \xi_4(t) \\
\frac{dz_5(t)}{dt} &= -\frac{3}{5} + \frac{1}{10(1+z_1(t))} + \frac{4z_2(t)}{5(1+z_2(t))} + \frac{3z_3(t)}{5(1+z_3(t))} + \frac{1}{10(1+z_4(t))} + \frac{1}{5(1+z_6(t))} + \frac{1}{5(1+z_7(t))} - \frac{7}{5}z_5(t) + \xi_5(t) \\
\frac{dz_6(t)}{dt} &= -\frac{1}{5} + \frac{z_2(t)}{5(1+z_2(t))} + \frac{z_3(t)}{5(1+z_3(t))} + \frac{1}{5(1+z_5(t))} + \frac{z_7(t)}{5(1+z_7(t))} - \frac{7}{5}z_6(t) + \xi_6(t) \\
\frac{dz_7(t)}{dt} &= -\frac{2}{5} + \frac{1}{5(1+z_2(t))} + \frac{1}{5(1+z_3(t))} + \frac{z_5(t)}{5(1+z_5(t))} + \frac{z_6(t)}{5(1+z_6(t))} - \frac{7}{5}z_7(t) + \xi_7(t) \\
\frac{dz_8(t)}{dt} &= -\frac{2}{5} + \frac{2z_1(t)}{5(1+z_1(t))} + \frac{2}{5(1+z_4(t))} - \frac{9}{5}z_8(t) + \xi_8(t) \\
\frac{dz_9(t)}{dt} &= -\frac{1}{2} + \frac{z_1(t)}{2(1+z_1(t))} + \frac{z_4(t)}{2(1+z_4(t))} - 2z_9(t) + \xi_9(t)
\end{aligned} \qquad (S1)$$

where p is a scalar control parameter and $\varsigma_i(t)(i = 1,2,\ldots,9)$ are Gaussian noises with zero means and covariances $k_{ij} = Cov(\varsigma_i, \varsigma_j)$. $z_i(t)(i = 1,2,\ldots,9)$ represent the concentrations of mRNA- i . In Eq.(S1), there is the degradation rates of mRNAs

R=$\left(\frac{10+3|P|}{20},\frac{3}{5},\frac{4}{5},\frac{10+3|P|}{20},\frac{7}{5},\frac{7}{5},\frac{7}{5},\frac{9}{5},2\right)$.The stable equilibrium point of the differential equations Eq.(S1) is $\overline{Z}=(\overline{z_1},\overline{z_2},\overline{z_3},\dots,\overline{z_{11}})=(0,0,0,\dots,0)$. The differential equations Eq.(S1) can be transformed into the difference equations $Z(k+1)=f(Z(k),p)$ using the Euler scheme [7] with a short time interval Δt. The result is as follows:

$$\begin{aligned}
z_1(k+1)&=z_1(k)+\left[\frac{(10-3|P|)z_4(t)}{20(1+z_4(t))}-\frac{10+3|P|}{20}z_1(t)+\xi_1(t)\right]\Delta k\\
z_2(k+1)&=z_2(k)+\left[\frac{(6-3|P|)z_1(t)}{20(1+z_1(t))}+\frac{(6-3|P|)z_4(t)}{20(1+z_4(t))}-\frac{3}{5}z_2(t)+\xi_2(t)\right]\Delta k\\
z_3(k+1)&=z_3(k)+\left[\frac{3|P|-8}{10}+\frac{8-3|P|}{20(1+z_1(t))}+\frac{8-3|P|}{20(1+z_4(t))}-\frac{4}{5}z_3(t)+\xi_3(t)\right]\Delta k\\
z_4(k+1)&=z_4(k)+\left[\frac{(10-3|P|)z_1(t)}{20(1+z_1(t))}-\frac{10+3|P|}{20}z_4(t)+\xi_4(t)\right]\Delta k\\
z_5(k+1)&=z_5(k)+\left[-\frac{3}{5}+\frac{1}{10(1+z_1(t))}+\frac{4z_2(t)}{5(1+z_2(t))}+\frac{3z_3(t)}{5(1+z_3(t))}+\frac{1}{10(1+z_4(t))}+\frac{1}{5(1+z_6(t))}+\frac{1}{5(1+z_7(t))}-\frac{7}{5}z_5(t)+\xi_5(t)\right]\Delta k\\
z_6(k+1)&=z_6(k)+\left[-\frac{1}{5}+\frac{z_2(t)}{5(1+z_2(t))}+\frac{z_3(t)}{5(1+z_3(t))}+\frac{1}{5(1+z_5(t))}+\frac{z_7(t)}{5(1+z_7(t))}-\frac{7}{5}z_6(t)+\xi_6(t)\right]\Delta k\\
z_7(k+1)&=z_7(k)+\left[-\frac{2}{5}+\frac{1}{5(1+z_2(t))}+\frac{1}{5(1+z_3(t))}+\frac{z_5(t)}{5(1+z_5(t))}+\frac{z_6(t)}{5(1+z_6(t))}-\frac{7}{5}z_7(t)+\xi_7(t)\right]\Delta k\\
z_8(k+1)&=z_8(k)+\left[-\frac{2}{5}+\frac{2z_1(t)}{5(1+z_1(t))}+\frac{2}{5(1+z_4(t))}-\frac{9}{5}z_8(t)\right]\Delta k\\
z_9(k+1)&=z_9(k)+\left[-\frac{1}{2}+\frac{z_1(t)}{2(1+z_1(t))}+\frac{z_4(t)}{2(1+z_4(t))}-2z_9(t)+\xi_9(t)\right]\Delta k
\end{aligned}\tag{S2}$$

It is easy to note that $Z(k)$ is the vector of $Z(t)$ at the time instant $k\Delta t$. The Jacobian matrix of Eq.(S2) can be defined as $J=\frac{\partial f(Z(k);p)}{\partial Z}\Big|_{Z=\overline{Z}}$, where

$$J=e^{\Delta t\bullet A}\tag{S3}$$

With

$$A=\begin{bmatrix} -\frac{10+3i}{20} & 0 & 0 & \frac{10-3i}{20} & 0 & 0 & 0 & 0 & 0 \\ \frac{6-3i}{20} & -\frac{3}{5} & 0 & \frac{6-3i}{20} & 0 & 0 & 0 & 0 & 0 \\ -\frac{8-3i}{20} & 0 & -\frac{4}{5} & -\frac{8-3i}{20} & 0 & 0 & 0 & 0 & 0 \\ \frac{10-3i}{20} & 0 & 0 & -\frac{10+3i}{20} & 0 & 0 & 0 & 0 & 0 \\ -\frac{1}{10} & \frac{4}{5} & \frac{3}{5} & -\frac{1}{10} & -\frac{7}{5} & -\frac{1}{5} & -\frac{1}{5} & 0 & 0 \\ 0 & \frac{1}{5} & \frac{1}{5} & 0 & -\frac{1}{5} & -\frac{7}{5} & -\frac{1}{5} & 0 & 0 \\ 0 & -\frac{1}{5} & -\frac{1}{5} & 0 & \frac{1}{5} & -\frac{1}{5} & -\frac{7}{5} & 0 & 0 \\ \frac{2}{5} & 0 & 0 & -\frac{2}{5} & 0 & 0 & 0 & -\frac{9}{5} & 0 \\ \frac{1}{2} & 0 & 0 & -\frac{1}{2} & 0 & 0 & 0 & 0 & -2 \end{bmatrix}$$

From Eq.(S3), by taking $\Delta t = 1$, we can obtain nine distinct eigenvalues $(0.74|P|,\ 0.55,\ 0.45,\ 0.37,\ 0.30,\ 0.25,\ 0.20,\ 0.17,\ 0.14)$ by taking $\Delta t = 1$. It is obvious that the largest eigenvalue $0.83^{|p|}$→1 when p→0. Therefore, the equilibrium point $\overline{Z}$ is stable when $p \in (0,1]$ and $p = 0$ is a bifurcation point, at which the system undergoes a critical transition. We aimed to detect early-warning signals that indicate the critical transition as a control parameter p approaches a bifurcation point 0. According to CPMI method, for each simulation trial, we used the 29 samples generated when the control parameter p was far away from the bifurcation point p = 0 (e.g., $p \in [-0.50, 0.25]$) as the reference samples. Then in each simulation trial, based on the single sample $\{z_1, z_2, \dots, z_{29}\}$ derived for each parameter value $p \in [-0.5, 0.25]$, the CPMI score was calculated following the CPMI method (see Method section in the main text), as shown in Figure 2 in the main text.

B. Critical state of mESC-to-MP

For mESC-to-MP data [8],it is seen from Figure S2 A, The CPMI score increased significantly and peaked at 24 h, indicating that a shift in cell fate was about to occur after 24 h, which preceded the induction of differentiation to endoderm at 48 h. The landscape evolution of the data is shown in Figure S2 B, demonstrating a significant increase in critical state scores during cell differentiation,the critical state is recognized before cell differentiation occurs. Figure S2 C shows the dynamic evolution of the DNB genes building protein-protein interaction network for the mESC-to-MP data, with a significant change in network structure at 24 hours, indicating an impending shift in the fate of cell differentiation. Then in Figure S2 D we used Ingenuity Pathway Analysis (IPA) to predict potential upstream transcriptional regulators of DNB genes during the critical stage. Transcription factors (TFs) are important players in defining cell identity and driving cell fate transitions [9]. We identified the top 20 transcription factors at the tipping point (24 h), the group of factors could regulate 78.7% of the DNB genes at the tipping point. In the predicted top 20 transcription factors, we found that ZNF207 plays an important role in the stability of cell fate and the maintenance of the differentiated state, thus providing a theoretical basis for understanding the molecular mechanisms of cell fate transitions [10]. Functional analysis shows that these regulated DNB genes are mainly involved in fluid shear stress and atherosclerosis, Antigen processing and presentation, tyrosine metabolism.(Figure S2 E).

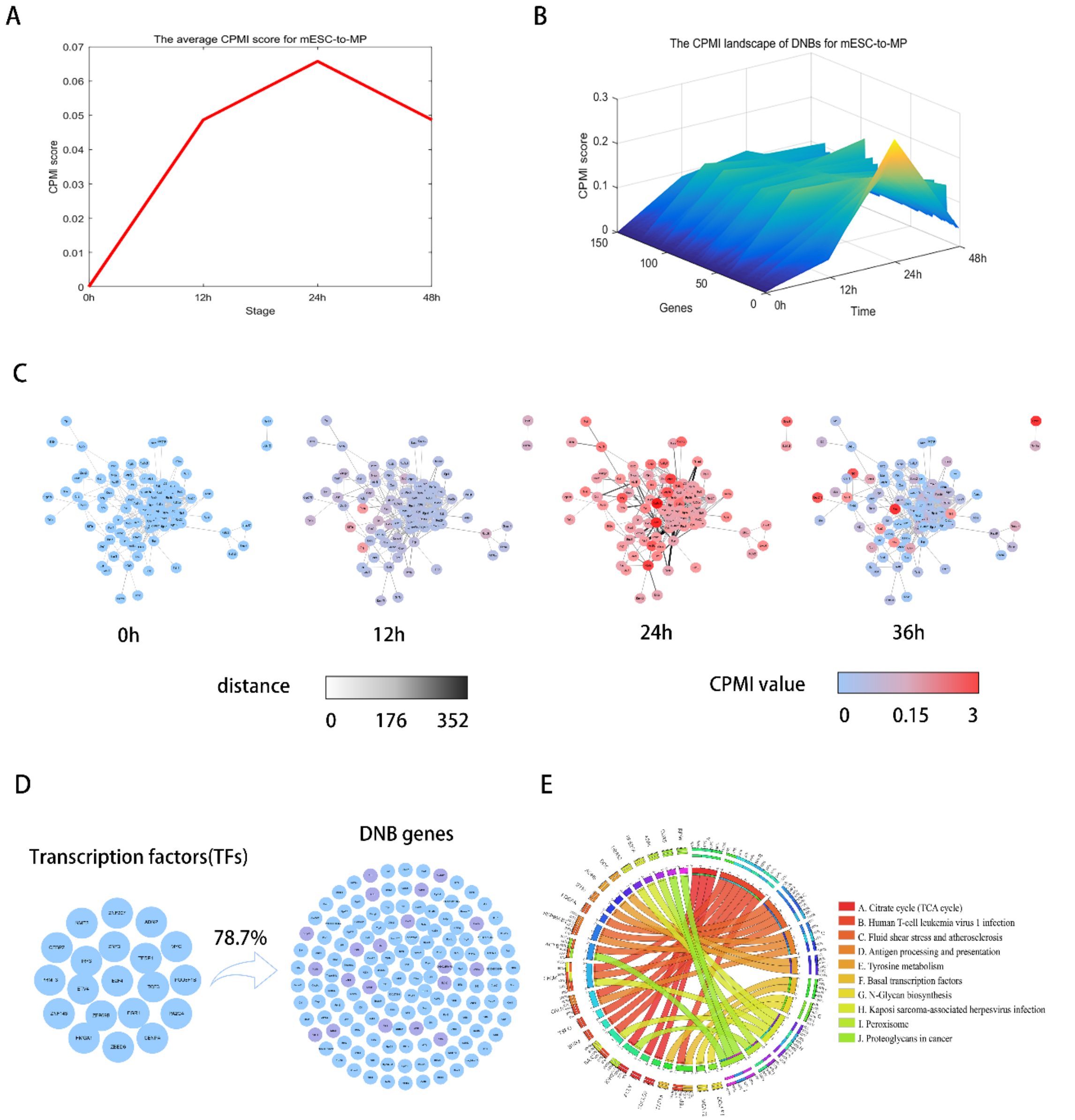

Figure S2. Identification of the cell fate transition of cell differentiation in mESC-to-MP dataset.(A)The CPMI score curve of mESC-to-MP. The significant increase of CPMI score at 24h indicates the arrival of the critical point.(B)The CPMI landscape of DNBs for mESC-to-MP. The overall CPMI score for the DNB gene is significantly higher at 24h than at other time points, indicating that it is in a critical state.(C)The dynamic evolution of DNBs for mESC-to-MP. At 24h, a significant change in the network shows that a critical warning signal can be detected. (D) The top20 hub upstream transcription factor, which regulates 78.7% of the DNB genes that were identified at 24h.(E) Enrichment analysis of regulated DNB genes during the mESC-to-MP process.

C. Critical state of THCA and COAD

For the THCA dataset [11], 58 tumor-adjacent samples and 381 tumor samples were obtained from TCGA. The CPMI score of THCA data(Figure S3 A) showed a significant change, with a sudden increase in stage II indicating that the disease began to worsen after this stage. This is consistent with the fact that cancer cells spread to the tissues and organs around the thyroid, and spread to other parts of the body or other organs through the blood circulation or Lymphatic system [12,13]. The deterioration of these diseases usually occurs in stage III [14,15]. As shown in Figure S3 B, the dynamic landscape of CPMI scores for stage II is significantly higher than for other stages, providing the early warning signals before disease deterioration. Figure S3 C depicts the comparison of THCA survival curves before and after the critical state. Before stageII, THCA has a longer survival time, and a higher probability of survival. Therefore, the identification of the critical state is important for the treatment and prognosis of cancer [16,17]. All of the above results verify that stageII is the critical state. In addition, The gene expression levels of DNBs at each stage are shown in Figure S3 G, and the average gene expression levels of DNBs at each stage are shown in Figure S3 H. It shows that the critical state and other states cannot be identified using simple gene expression method, while using our CPMI method can effectively detect the critical state before disease progression occurs.

For the COAD dataset [11], 35 tumor-adjacent samples and 155 tumor samples were obtained from TCGA. In Figures S3 D,the CPMI score peak at stageII, suggesting an impending critical transition. Indeed, after stageII, the adenocarcinoma cells begin to invade the mucosa and submucosa of the colon and may spread to the adjacent lymph nodes [12]. In addition, the cancer cells further invade the muscularis and plasma membrane of the colon wall and spread to adjacent tissues and organs, such as the spleen and bladder, which generally occur at stage III [18]. As shown in Figure S3 E, a marked increase of the CPMI score at stageII, suggesting the imminent arrival of a critical transition.In Figure S3 F, there was a significant difference in the survival curves for COAD before and after stageII, i.e. samples before the critical state survived longer than those after the critical state.

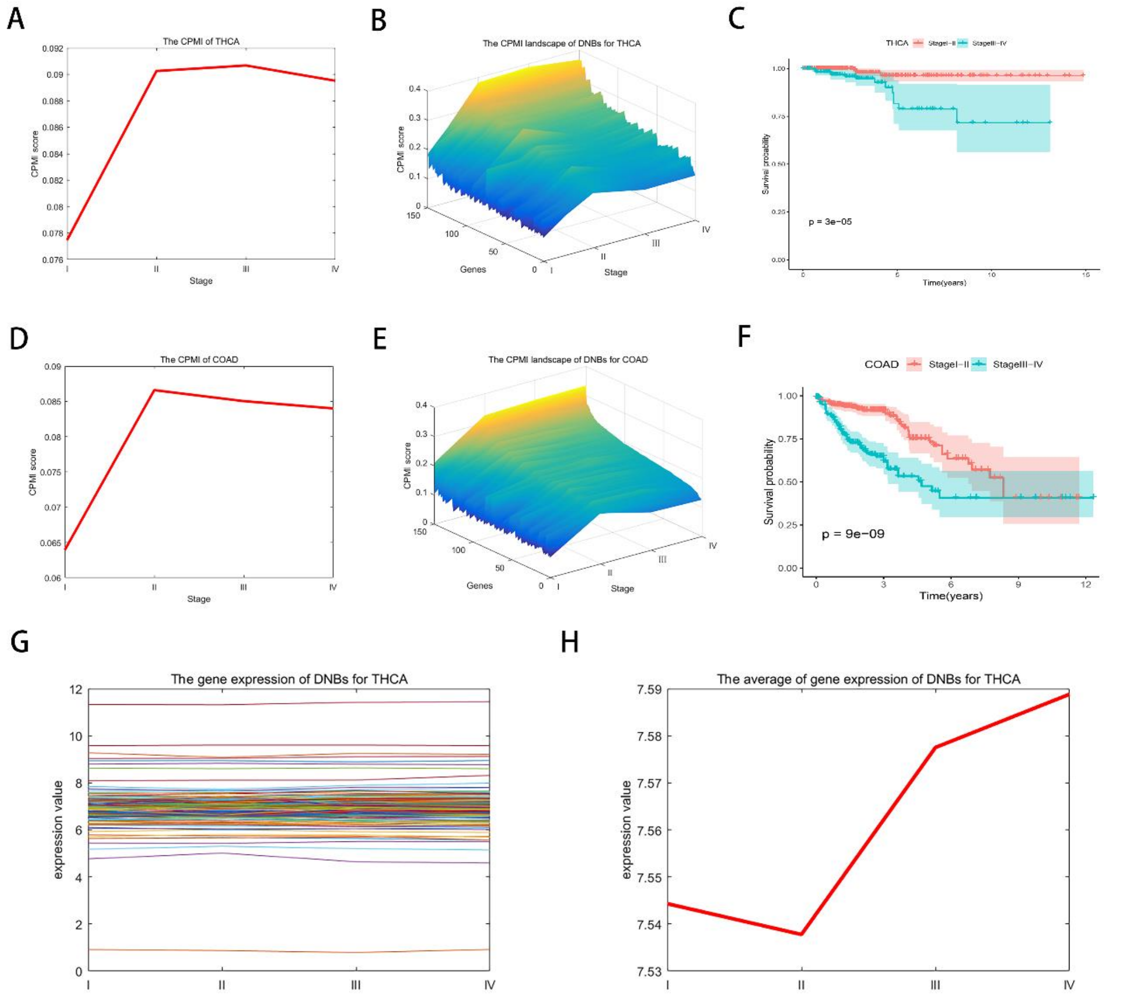

Figure S3. Identification of critical stages for cancer metastasis in THCA and COAD. (A)The CPMI score curve of THCA. (B)The CPMI landscape of DNBs for THCA.(C) The comparison of THCA survival curves before and after the critical state. (D)The CPMI score curve of COAD. (E)The CPMI landscape of DNBs for COAD. (F) The comparison of COAD survival curves before and after the critical state.(G)The gene expression of DNBs for THCA.(H)The average of gene expression of DNBs for THCA.

D. The analysis of ‘dark genes’ for hESC-to-DEC and KIRP

We will elaborate on the contribution of dark genes to complex biological processes from two aspects and have revised the original text accordingly. We find that many of these genes play important roles in complex biological progression by functional analysis, although they are usually ignored in the traditional differential expression analysis.

(1) The prognosis analysis of ‘dark genes’

In order to study their biological meaning during complex biological process, we make prognosis analysis for these ‘dark genes’ based on patients’ clinical information. We divide the samples into high-score group and the low-score group based on the median of gene expressions. Through survival curves based on the gene expressions, we find that there is an obvious difference in the survival curve. Therefore, the “dark genes” can be used as key indicators for early diagnosis of disease deterioration.

(2) The functional analysis of ‘dark genes’

The functional analysis shows that the ‘dark genes’ are enriched into the key biological processes or functions associated with the development of complex biological. For example, as shown in Figure S4A, the ‘dark genes’ (HSP90AB1, ITGAV, YWHAZ and CKS2) in hESC-to-DEC are enriched into signaling pathways of cell differentiation or cell regulation, e.g., PI3K-Akt signaling pathway (hsa04151), Pathways in cancer (hsa05200), Small cell lung cancer (hsa05222) and Fluid shear stress and atherosclerosis (hsa05418). Besides, as shown in Figure S4B, the ‘dark genes’(ITGB1, JUP, GNB1 and RHOA) in KIRP are enriched in pathways closely associated with the development of kidney papillary cell carcinoma, e.g., pathways in cancer and PI3K-Akt signaling pathway. The ‘dark genes’ (ITGB1, ARF3, GPI, JUP, NUMA1, RHOA, CNDP2, HINT1, TMBIM1, CLTCL1, GNB1, CAPN2, ADD1 and CPVL) are enriched into biological processes of cellular signal transduction, cell adhesion and intercellular communication including protein binding (GO:0005515), extracellular exosome (GO:0070062) , macromolecular complex binding (GO:0044877) and focal adhesion (GO:0005925).

During the development of cancer, abnormal biological processes such as aberrant protein binding, extracellular vesicle release, abnormal changes in cytoplasm and cell membrane, and alterations in focal adhesions may occur, promoting the growth, spread, and metastasis of cancer cells. Through literature searching, we find that some ‘dark genes’ are closely related to the process of complex biological. For example, in hepatocellular carcinoma (HCC), GNB1 can serve as a prognostic biomarker for HCC patients, and the prognosis is poorer in liver cancer patients with GNB1 overexpression. Overexpressing GNB1 exerted oncogenic functions via the interaction of BAG2 and activated P38 signaling pathway [19]. Diffuse gastric

cancer (DGC) patients with higher expressed of ITGB1 had poor prognosis, while reduced expression of ITGB1 inhibited the ability of cell proliferation and motility [20]. Overexpression of ZNF521 significantly reduces osteoblast differentiation and inhibits adipogenic differentiation [21].

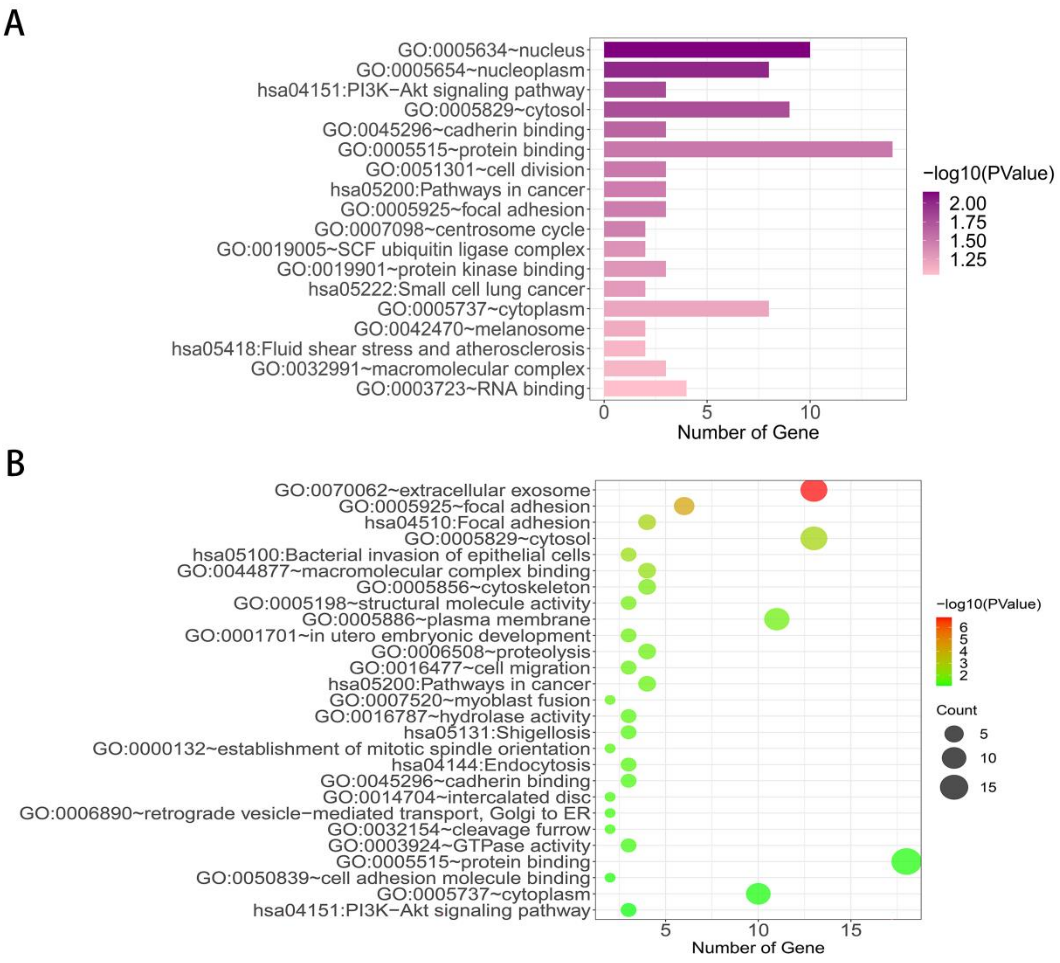

Figure S4. The functional analysis of ‘dark genes’ for hESC-to-DEC and KIRP. A hESC-to-DEC dataset. B KIRP dataset.

E. The analysis of ‘dark genes’ for mESC-to-MP

E1. The expression analysis of “dark genes” for mESC-to-MP

As shown in Figure S5, ACLY, ATPIF1,BTF3, CACYBP, MGAT2 and RP17A are found to be ‘dark genes’. We have compared the changes in CPMI scores and gene expression of ‘dark genes’ at each time point. The CPMI score of ‘dark genes’ becomes more sensitive when the cell fate transition comes and shows obvious upward trends before the differentiation induction into mesoderm progenitor (MP) at 24 h compared to the gene expression data.

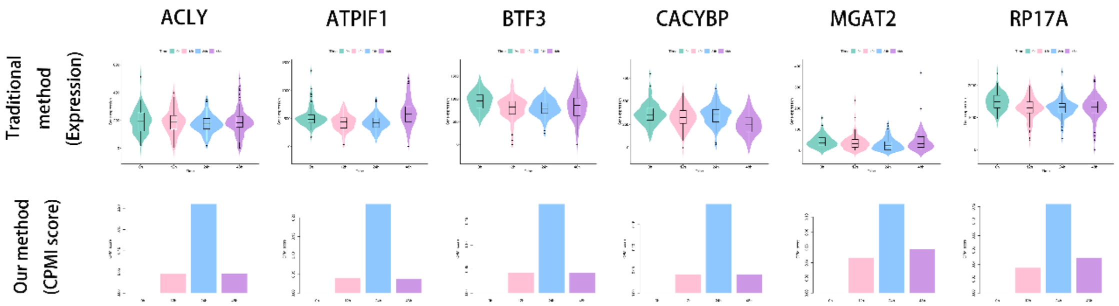

Figure S5. The comparison of gene expression and CPMI score of ‘dark genes’ for mESC-to-MP, ACLY, ATPIF1,BTF3, CACYBP, MGAT2 and RP17A, are found to be dark genes of mESC-to-MP, whose CPMI scores are more sensitive to the early warning signal of embryonic differentiation.

E2. The functional analysis of “dark genes” for mESC-to-MP

The functional analysis demonstrates that some ‘dark genes’ (IDH2, TSPO, RPS19, RPS6, ACLY, DDC, MCEE, GSTM1, MPI, IDH2, PPT1, SUCLG2, MGAT2, ADSS, ADH5, ATP6V0E and PRPSAP2) in mESC-to-MP are enriched in biological processes related to cell metabolism, cell proliferation and apoptosis. For instance protein transport (GO:0015031), Metabolic pathways (mmu:01100), negative regulation of glial cell proliferation (GO: 0060253), ribosomal small subunit assembly (GO: 0000028) and purine nucleotide biosynthetic process (GO: 0006164). In addition,some ‘dark genes’ are of great importance in the process of cell differentiation of mESC-to-MP, such as ATPIF1, ACLY and CACYBP. Overexpression of ATPIF1 attenuates changes in cell proliferation, adhesion and migration induced by a number of factors, as well as helps to maintain the normal structure of mitochondria [22]. The reduced expression of ACLY can decreased the cell viability, inhibited the cell proliferation, invasion and metastasis, and induced the cell apoptosis. In addition, ACLY has great potential to become a key target for cancer therapy [23]. CACYBP is involved in the process of cell differentiation and proliferation by regulating the Akt signalling pathway, and is also the participant in a variety of other biological processes, and its gene expression is strongly correlated with the level of patient prognosis.

F. The functional analysis for hESC-to-DEC and mESC-to-MP

F1. The functional analysis for hESC-to-DEC

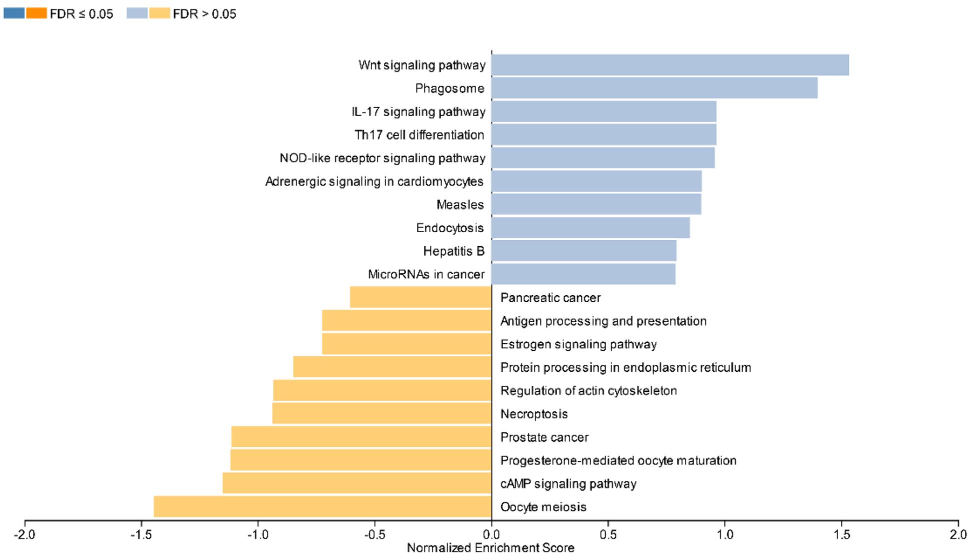

Figure S6. GSEA enrichment analysis of DNBs for hESC-to-DEC. The enriched pathways included Wnt signaling pathway, Th17 cell differentiation and cAMP signaling pathway.

Table S1. The functional enrichment of DNBs in the critical state samples for hESC-to-DEC

Gene Ontology Consortium		KEGG	
enriched biological process	enriched p value	enriched biological process	enriched p value
anterior/posterior axis specification (GO: 0009948)	2.40E-04	Small cell lung cancer (hsa05222)	0.0053371
negative regulation of transcription from RNA polymerase II promoter (GO: 0000122)	6.65E-04	PI3K-Akt signaling pathway (hsa04151)	0.0182762
chaperone-mediated autophagy (GO: 0061684)	8.59E-04	MicroRNAs in cancer (hsa05206)	0.0310854
positive regulation of transforming growth factor beta receptor signaling pathway (GO: 0030511)	0.001379	Prostate cancer (hsa05215)	0.0382334
positive regulation of tau-protein kinase activity (GO: 1902949)	0.0014602	NOD-like receptor signaling pathway(hsa04621)	0.0542218
negative regulation of apoptotic process (GO: 0043066)	0.0021578	Oxidative phosphorylation (hsa00190)	0.0835089
negative regulation of canonical Wnt signaling pathway (GO: 0090090)	0.0049369	Fluid shear stress and atherosclerosis(hsa05418)	0.0908561
positive regulation of cell migration (GO: 0030335)	0.0073103	Chemical carcinogenesis - reactive oxygen species (hs 05208)	0.0916706

F2. The functional analysis for mESC-to-MP

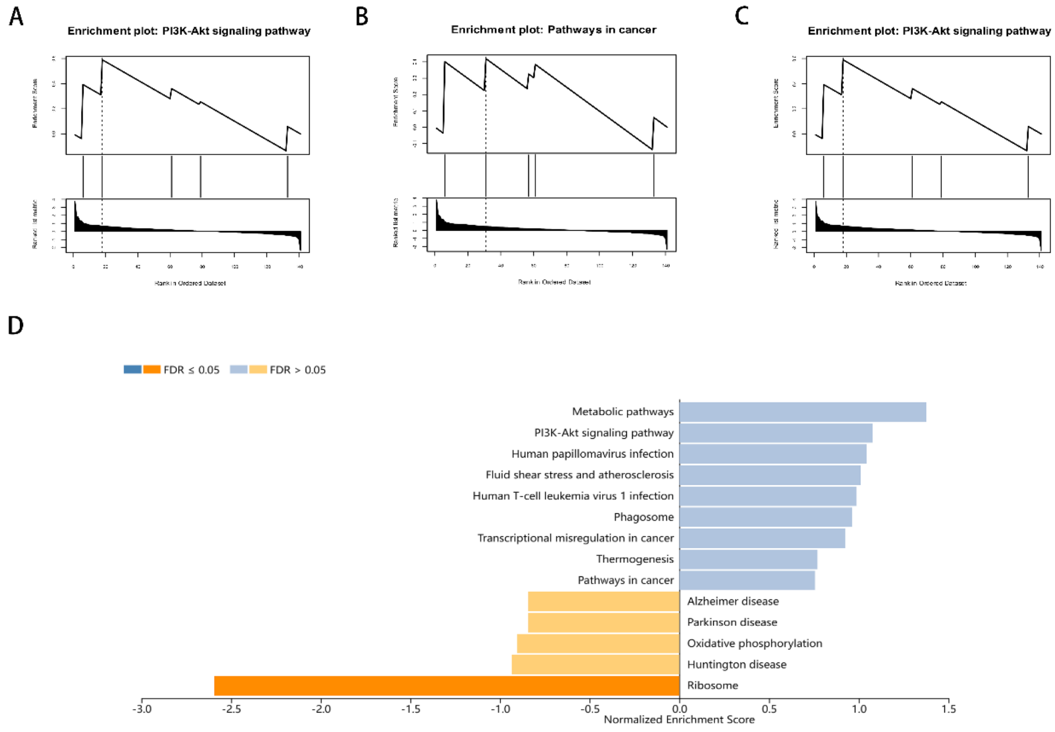

Figure S7. GSEA enrichment analysis of DNBs for mESC-to-MP. The enriched pathways included P13K-AKT signaling pathway, Pathway in cancer and Metabolic pathways.

Table S2. The functional enrichment of DNBs in the critical state samples for mESC-to-MP

Gene Ontology Consortium		KEGG	
enriched biological process	enriched p value	enriched biological process	enriched p value
cytoplasmic translation (GO:0002181)	1.53E-40	Ribosome (mmu03010)	1.54E-31
ribosomal small subunit biogenesis (GO:0042274)	2.13E-11	Coronavirus disease - COVID-19 (mmu05171)	2.54E-28
response to xenobiotic stimulus (GO:0009410)	0.0032699	Citrate cycle (TCA cycle) (mmu00020)	0.0319434
protein stabilization (GO:0050821)	0.0015197	Fluid shear stress and atherosclerosis (mmu05418)	0.0402623
positive regulation of cell proliferation (GO:0008284)	0.0166705	Human T-cell leukemia virus 1 infection (mmu05166)	0.0224567
ATP synthesis coupled proton transport (GO:0015986)	0.0085257	Antigen processing and presentation (mmu04612)	0.0445879
negative regulation of RNA splicing (GO:0033119)	0.0417779	Phagosome (mmu04145)	0.076065
positive regulation of apoptotic process (GO:0043065)	0.0114998	Carbon metabolism(mmu01200)	0.0903963

G. Validating the identified critical state by The Kaplan-Meier (log-rank) survival analysis

To further validate the plausibility of identifying the critical state, we also proposed the survival analyses on some samples at different stages and compared through Kaplan-Meier (log-rank) survival analysis. The survival analysis of samples before and after unidentified state can be found in Figure S8 and Figure S9. Specifically, by comparing the differences in survival before and after the critical state and other states, we determined that there is a longer survival time and a higher probability of survival before the critical state, while there was no significant differences before and after the other stages.

G1. Validating the identified critical state for THCA

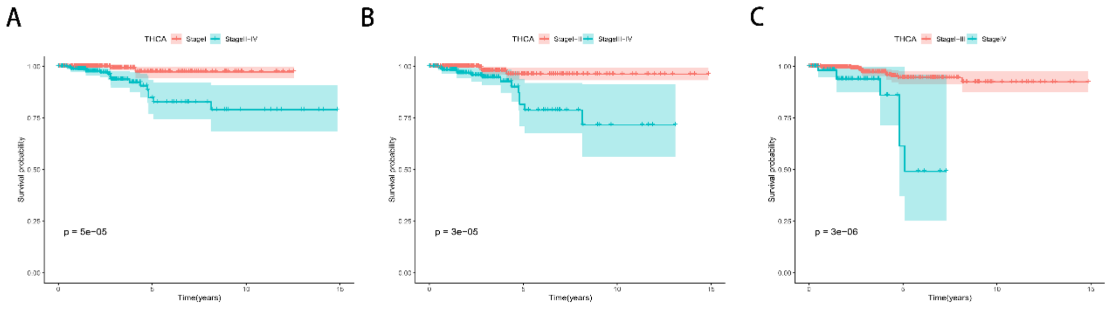

Figure S8. The comparison of survival times for THCA before and after every state. A. Stage I of THCA vs the stages after Stage I of THCA in survival analysis. B. The stage before stage II of THCA vs the stages after Stage II of THCA in survival analysis. C. The stages before stage III of THCA vs the stages after stage III of THCA in survival analysis.

G2. Validating the identified critical state for COAD

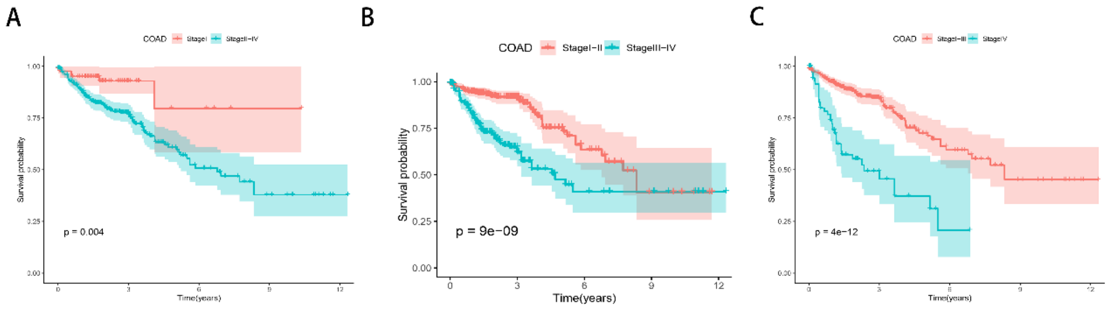

Figure S9. The comparison of survival times for COAD before and after every state. A. Stage I of COAD vs the stages after Stage I of COAD in survival analysis. B. The stage before stage II of COAD vs the stages after Stage II of COAD in survival analysis. C. The stages before stage III of COAD vs the stages after stage III of COAD in survival analysis.

H. The functional analysis for THCA and COAD

H1. The functional analysis for THCA

Table S3. The functional enrichment of DNBs in the critical state samples for THCA

Gene Ontology Consortium		KEGG	
enriched biological process	enriched p value	enriched biological process	enriched p value
dolichol-linked oligosaccharide biosynthetic process (GO:0006488)	6.23E-06	N-Glycan biosynthesis (hsa00510)	2.12E-06
protein glycosylation (GO:0006486)	9.22E-05	Various types of N-glycan biosynthesis (hsa00513)	2.37E-04
intracellular protein transport (GO:0006886)	1.99E-04	Metabolic pathways (hsa01100)	0.0118922
cell cycle (GO:0007049)	4.19E-04	Shigellosis (hsa05131)	0.0091178
autophagosome assembly (GO:0000045)	7.44E-04	Autophagy - other (hsa04136)	0.0015953
regulation of small GTPase mediated signal transduction (GO:0051056)	0.0063864	Mitophagy - animal (hsa04137)	0.0155959
negative stranded viral RNA replication (GO:0051897)	0.0184299	Longevity regulating pathway (hsa04211)	0.0272165
positive regulation of myoblast differentiation (GO:0045663)	0.0328596	Progesterone-mediated oocyte maturation (hsa04914)	0.0385063

H2. The functional analysis for COAD

Table S4. The functional enrichment of DNBs in the critical state samples for COAD

Gene Ontology Consortium		KEGG	
enriched biological process	enriched p value	enriched biological process	enriched p value
protein folding in endoplasmic reticulum (GO:0034975)	8.22E-07	Protein processing in endoplasmic reticulum (hsa04141)	2.25E-07
negative regulation of apoptotic process (GO:0043066)	1.18E-06	Antigen processing and presentation (hsa04612)	4.78E-07
response to endoplasmic reticulum stress (GO:0034976)	2.49E-06	NOD-like receptor signaling pathway (hsa04621)	5.16E-04
endoplasmic reticulum unfolded protein response (GO:0030968)	3.27E-05	Cycolysis / Gluconeogenesis (hsa00010)	0.001461
epithelial cell differentiation (GO:0030855)	9.13E-06	Human T-cell leukemia virus 1 infection (hsa05166)	0.0018067
positive regulation of protein import into nucleus (GO:0042307)	2.57E-04	Thyroid hormone synthesis (hsa04918)	0.0024129
positive regulation of protein kinase B signaling (GO:0051897)	3.47E-04	Parkinson disease(hsa05012)	0.0060052
positive regulation of nitric oxide biosynthetic process (GO:0045429)	3.34E-04	HIF-1 signaling pathway(hsa04066)	0.0117012

I. Leave-one-out cross validation

We carry out leave-one-out cross validation by identifying DNBs for KIRP (kidney renal papillary cell carcinoma), the number of selected genes in these validations is 5% of the total number of genes. The procedure is as follows:

(1) For KIRP dataset, leaving a case sample $X_i, i = 1, 2, \ldots \ldots N$ out, we used the remaining N-1 case samples for identifying the DNBs based on the CPMI, and then calculated its CPMI score at each stage, i.e., $CPMI_i, i = 1, 2, \ldots \ldots N$, where N represents the number of case samples.

(2) By using the identified DNBs, we carried out the validation for the excluded sample based on the CPMI score. Finally, the average CPMI with the leave-one-out cross validation was obtained by $\sum_{i=1}^{N} CPMI_i / N$.

Following the above procedure, we obtained N identified DNBs, and N validation results respectively for KIRP dataset. In which the CPMI scores validated our previous results presented in Fig. 6 of the main text. The total number of samples used to identify DNBs was 25. In order to avoid the potential problem of the submission system shutting down and preventing us from submitting revised manuscripts, we randomly selected 7 samples from the 25 case samples for cross validation. As shown in Figure S10, where red curves represent the average CPMI of the identified DNBs with all samples, and the blue curves represent the average CPMI of the identified DNBs with leave-one-out cross validation. It can be seen that the average CPMI curves with leave-one-out cross validation have the same trends to those of the DNBs with all samples, which validates the effectiveness of the detected critical state.

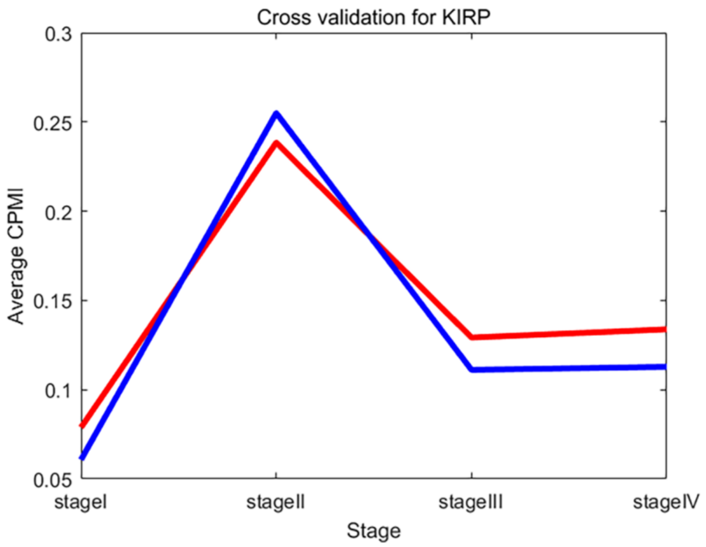

Figure S10. Cross validation results for KIRP dataset. The red curves represent the average CPMI of the identified DNBs with all samples, and the blue curves represent the average CPMI of the identified DNBs with leave-one-out cross validation.

J. The discussion on the Mahalanobis distance denominator

We have explained the reasons for using asymmetric distance measures in model construction from three aspects. Additionally, we have compared our model with one that is normalized by the product of s_i and s_j.

(1) In biological systems, the regulatory relationships between genes are often asymmetric. While one gene may regulate another gene, the reverse may not necessarily be true. The use of asymmetric distances can better reflect this biological asymmetry, thus more accurately simulating biological processes.

(2) Additionally, gene expression data typically exhibit different statistical characteristics, such as varying variances and distributions. Asymmetric distances allow for the consideration of the standard deviation of each gene separately, aiding in revealing important patterns in gene expression level variations.

(3) Furthermore, gene expression datasets often exhibit heterogeneity, and asymmetric distances can help in handling the heterogeneity between different genes, for example by selecting nearest neighbor genes with specificity in a local network centered on gene i. Moreover, compared to the standard Mahalanobis distance which normalizes distances based on the covariance matrix between two samples, using asymmetric distance metrics in model construction can enhance computational efficiency, reducing runtime and costs. While our algorithm does have certain limitations, we plan to make modifications to it in the future.

In the model construction, we compared two different methods: one using asymmetric distance measures and the other normalized by the product of s_i and s_j. We evaluated the performance of these two models on a dataset generated from numerical simulations. As shown in Figure S11, the red curve represents our model, denoted as CPMI1, the blue curve represents the model normalized by the product of s_i and s_j, denoted as CPMI2. Under different noise strengths, our model outperforms the model using the s_i and s_j product normalization method in identifying the critical state.

In addition, We evaluated the performance of these two models on a real data, i.e., influenza dataset. We selected three participants for the study using the influenza dataset. As shown in Figure S12, subject 1, subject 5 and subject 6 with influenza symptoms in our model exhibit a significant increase in CPMI score (red curve) before the onset of influenza symptoms, providing early warning signals for imminent critical transitions. In contrast, the CPMI score of another model (blue curve) exhibits no notable change. So our model outperforms the model using s_i and s_j product normalization method in identifying the critical state.

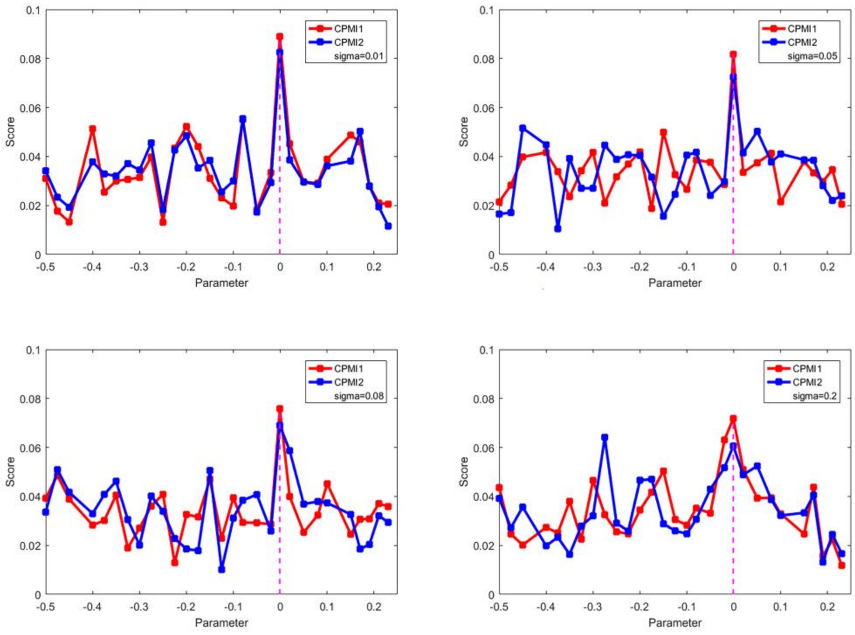

Figure S11. Comparison of the robustness of the CPMI method with the model normalized by the product of s_i and s_j method at different noise strength. The red curve represents our model, denoted as CPMI1, the blue curve represents the model normalized by the product of s_i and s_j, denoted as CPMI2.

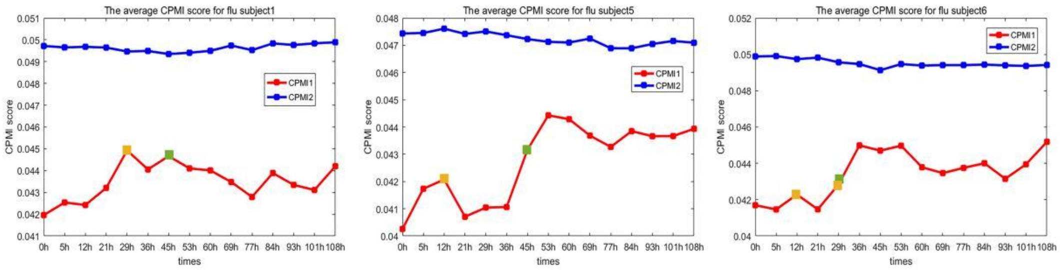

Figure S12. Comparison of the performance of the CPMI method on the influenza dataset individual 1, 5 and 6 with the model normalized by the product of s_i and s_j method. The red curve represents our model, denoted as CPMI1, the blue curve represents the model normalized by the product of s_i and s_j, denoted as CPMI2. The green box represents the initial time to the appearance of influenza symptoms (clinical observation), and the orange box represents the critical state determined by the CPMI score.

References

1. Khanin R, Vinciotti V, Mersinias V, et al. Statistical Reconstruction of Transcription Factor Activity Using Michaelis–Menten Kinetics. Biometrics 2007; 63:816-823.
2. Ronen M, Rosenberg R, Shraiman B, et al. Assigning numbers to the arrows: parameterizing a gene regulation network by using accurate expression kinetics. Proc Natl Acad Sci U S A 2002; 99(16):10555-10560.
3. Sueyoshi C, Naka T. Stability Analysis for the Cellular Signaling Systems Composed of Two Phosphorylation-Dephosphorylation Cyclic Reactions. Computational Molecular Bioscience 2017; 7:33-45.
4. Chen L, Wang R, Li C, et al. Modeling Biomolecular Networks in Cells: Structures and Dynamics. Springer London 2010.
5. Chen L, Wang R, Zhang X. Biomolecular Networks: Methods and Applications in Systems Biology. John Wiley & Sons, Inc 2009; DOI: 10.1002/9780470488065.
6. Chen P, Li Y, Liu X, et al. Detecting the tipping points in a three-state model of complex diseases by temporal differential networks. J Transl Med 2017; 15(1):217.
7. Fong, A.P., and Tapscott, S.J. (2013). Skeletal muscle programming and re-programming. Curr. Opin. Genet. Dev. 23, 568–573.
8. Semrau S, Goldmann JE, Soumillon M, Mikkelsen TS, Jaenisch R, van Oudenaarden A. Dynamics of lineage commitment revealed by single-cell transcriptomics of differentiating embryonic stem cells. Nat Commun 2017;8:1096.
9. Takahashi, K., and Yamanaka, S. (2016). A decade of transcription factor-mediated reprogramming to pluripotency. Nat. Rev. Mol. Cell Biol. 17, 183–193.
10. Missinato MA, Murphy S, Lynott M, Yu MS, Kervadec A, Chang YL, Kannan S, Loreti M, Lee C, Amatya P, Tanaka H, Huang CT, Puri PL, Kwon C, Adams PD, Qian L, Sacco A, Andersen P, Colas AR. Conserved transcription factors promote cell fate stability and restrict reprogramming potential in differentiated cells. Nat Commun. 2023 Mar 27;14(1):1709. doi: 10.1038/s41467-023-37256-8.
11. Peng H, Zhong J, Chen P, Liu R. Identifying the critical states of complex diseases by the dynamic change of multivariate distribution. Brief Bioinform. 2022 Sep 20;23(5):bbac177.
12. Bareschino MA, Schettino C, Rossi A, et al.Treatment of advanced non small cell lung cancer. J Thorac Dis 2011;3:122.
13. Shaha AR. TNM classification of thyroid carcinoma. World J Surg 2007;31:879–87.
14. Li J,Wang J, Xie D, et al.Characteristics of the PI3K/AKT and MAPK/ERK pathways involved in the maintenance of self-renewal in lung cancer stem-like cells. Int J Biol Sci 2021;17:1191.

15. Twelves C, Wong A, Nowacki MP, et al. Capecitabine as adjuvant treatment for stage III colon cancer. N Engl J Med 2005;352: 2696–704.
16. Gasent Blesa JM, Grande Pulido E, Provencio Pulla M, et al. Old and new insights in the treatment of thyroid carcinoma. J Thyroid Res 2010;2010:1–16.
17. Cheung DY, Kim JK. Perspectives of the stomach cancer treatment: the introduction of molecular targeted therapy and the hope for cure. Korean J Gastroenterol 2013;61:117–27.
18. Hari DM, Leung AM, Lee J-H, et al. AJCC Cancer staging manual 7th edition criteria for colon cancer: do the complex modifications improve prognostic assessment? J Am Coll Surg 2013;217: 181–90
19. Zhang X, Dong K, Zhang J, Kuang T, Luo Y, Yu J, Yu J, Wang W. GNB1 promotes hepatocellular carcinoma progression by targeting BAG2 to activate P38/MAPK signaling. Cancer Sci. 2023 May;114(5):2001-2013. doi: 10.1111/cas.15741.
20. Zhu X, Chen H, Li H, Ren H, Ye C, Xu K, Liu J, Du F, Zhang Z, Liu Y, Xie X, Wang M, Ma T, Chong W, Shang L, Li L. ITGB1-mediated molecular landscape and cuproptosis phenotype induced the worse prognosis in diffuse gastric cancer. Front Oncol. 2023 Mar 16;13:1115510. doi: 10.3389/fonc.2023.1115510.
21. Chiarella E, Aloisio A, Scicchitano S, Lucchino V, Montalcini Y, Galasso O, Greco M, Gasparini G, Mesuraca M, Bond HM, Morrone G. ZNF521 Represses Osteoblastic Differentiation in Human Adipose-Derived Stem Cells. Int J Mol Sci. 2018 Dec 18;19(12):4095. doi: 10.3390/ijms19124095.
22. Wang K, Chen H, Zhou Z, Zhang H, Zhou HJ, Min W. ATPIF1 maintains normal mitochondrial structure which is impaired by CCM3 deficiency in endothelial cells. Cell Biosci. 2021 Jan 9;11(1):11.
23. Granchi C. ATP citrate lyase (ACLY) inhibitors: An anti-cancer strategy at the crossroads of glucose and lipid metabolism. Eur J Med Chem. 2018 Sep 5;157:1276-1291.
